# A Longitudinal Study of Multidimensional Prosocial Behavior During Adolescence

**DOI:** 10.1111/cdev.70009

**Published:** 2025-07-25

**Authors:** Sophie W. Sweijen, Lysanne W. te Brinke, Suzanne van de Groep, Eveline A. Crone

**Affiliations:** ^1^ Erasmus School of Social and Behavioral Sciences Erasmus University Rotterdam Rotterdam the Netherlands

**Keywords:** adolescence, prosocial behavior, risk‐taking behavior

## Abstract

This study examines the distinct developmental trajectories of prosocial and rebellious behaviors in adolescence. Using data from an accelerated three‐wave project (2018–2022) among adolescents aged 9–22 years (*N* = 142, 63% female, middle‐high SES, white European descent), trajectories of prosocial actions toward friends and peers, prosocial tendencies across multiple situations, giving to charities, and general social value orientation were examined. By examining age‐, puberty‐, and hormonal‐related trajectories, the study demonstrates increases in prosocial behaviors to friends and peers, dire and compliant behavior, and charitable giving, which were more strongly explained by pubertal maturation than age. Public prosocial behavior decreased with age. The results confirm the multidimensionality of prosocial behavior, demonstrate correlations with rebelliousness, and show that prosocial behavior is context‐dependent.

Prosocial behaviors, which are defined as behaviors intended to benefit others, are important building blocks for developing and maintaining positive social relationships (Carlo and Padilla‐Walker 2020). Adolescence, the period between ages 10 and 24 years (Sawyer et al. 2018), is characterized by age‐related changes in prosocial behavior, with most studies reporting a general increase in prosocial behaviors across adolescence (e.g., Crone et al. 2022; Do et al. 2017). During this developmental period, adolescents undergo rapid biological, cognitive, and social transitions that shape how, when, and toward whom they act prosocially (Crone et al. 2022; Crone and Dahl 2012). Pubertal maturation, shifting peer dynamics, and increasing autonomy contribute to greater differentiation in prosocial behaviors (Crone et al. 2022). Adolescents navigate increasingly complex social environments with heightened focus on peer approval (Blakemore and Mills 2014; Güroğlu 2020), which makes adolescence a crucial period for the development of prosocial behavior that may co‐occur with risk‐taking (Do et al. 2017). Rather than following a single developmental path, prosocial behaviors become more context‐dependent and strategic (e.g., Güroğlu et al. 2014; Padilla‐Walker et al. 2018; van de Groep et al. 2022), making this period particularly valuable for examining how prosocial behaviors develop and how this development intersects with rebelliousness. However, the exact developmental patterns of prosocial behavior are still debated (Carlo and Padilla‐Walker 2020). The large heterogeneity across developmental studies, mainly depending on specific design parameters used, can be interpreted as evidence that prosocial behavior should be regarded as a multidimensional construct (Carlo and Padilla‐Walker 2020). According to this hypothesis, prosocial behavior comprises many behaviors, from sharing and helping to cooperating, and depends on several contextual characteristics (e.g., the beneficiary and specific situations, such as dire and public). As presented in the multidimensional model of prosocial behaviors by Carlo and Padilla‐Walker (2020), the three dimensions affecting prosocial behavior are the *context* (e.g., an anonymous or dire situation), *target* (e.g., an in‐group member of a stranger), and *motivation* (e.g., selfless or selfish). Particularly during adolescence, it is important to explore when, to whom, and why to show prosocial behavior. Therefore, these dimensions of prosocial behaviors should be considered when investigating their developmental trajectories, as each type of prosocial behavior may show differential patterns. The current study adopts a developmental lens to examine how prosocial behaviors unfold across different domains (e.g., peers and charities) and how these are shaped by age‐, puberty‐, and hormonal‐related trajectories. To capture these processes across early, mid‐, and late adolescence, we employed an accelerated longitudinal design with a wide age range (9–22 years), allowing us to efficiently model intra‐individual developmental change of prosocial behaviors and rebelliousness without large measurement gaps. Here, we focus on multiple types of prosocial behaviors, as measured by both self‐report questionnaires and experimental tasks. This way, we are able to assess individuals' general tendencies to show certain types of prosocial behaviors, as well as disentangle contextual factors that influence actual prosocial behaviors. This design choice reflects our focus on capturing meaningful variation across a broad, but developmentally coherent window in which prosocial and rebellious behaviors emerge and differentiate.

## 1 Development of Multidimensional Prosocial Behavior Across Adolescence

During adolescence, peers become more important as adolescents transition from caregiver dependency to independence (Crone and Dahl 2012), reinforcing the impact peers have on adolescents' prosocial decisions (Güroğlu 2020). Developmental studies have shown significant increases from early adolescence to young adulthood in prosocial behavior toward friends (Güroğlu et al. 2014; Padilla‐Walker et al. 2018). Non‐linear trajectories have also been reported. A cross‐sectional study by Blankenstein et al. (2020) showed a peak in prosocial actions to friends and peers during mid‐to‐late adolescence. Also, a cross‐sectional study during COVID‐19 demonstrated a peak in specifically emotional support to friends during late adolescence (Sweijen et al. 2022). On the basis of these previous studies, we examined developmental trajectories of different prosocial actions among adolescents toward their friends and peers, namely, altruism (i.e., prosocial behavior without direct personal gains), emotional support (i.e., providing comfort), helping (i.e., providing practical support), and giving/sharing (i.e., giving or sharing valuable resources).

Researchers have also argued that situational‐specific prosocial behaviors can be distinguished, such as public, anonymous, dire, emotional, compliant, and altruistic prosocial behaviors. Although general personal tendencies are suggested to increase with age (Eisenberg et al. 2006), differential patterns in prosocial tendencies between different age groups have been discussed. For example, Carlo et al. (2003) have shown that individuals during mid‐adolescence show more altruistic and anonymous prosocial behaviors than early adolescents, but the same does not apply to the other situations (i.e., public, dire, emotional, and compliant). Also, distinct associations have been found between the situational‐specific behaviors and several social and cultural processes that are known to develop throughout adolescence (Blakemore and Mills 2014; Carlo and Padilla‐Walker 2020), such as empathy (Azimpour et al. 2012; Rodrigues et al. 2017). However, to our knowledge, no longitudinal study has investigated developmental patterns of different prosocial behaviors within the same adolescents. In the current study, we therefore examined the developmental trajectories of public (i.e., in the presence of others), anonymous (i.e., without others' knowledge), dire (i.e., during crises), emotional (i.e., during emotional situations), compliant (i.e., when asked to), and altruistic prosocial behaviors across adolescence (Carlo et al. 2003).

To study prosocial behavior while being less dependent on self‐report, researchers have made use of the Dictator Game (Eckel and Grossman 1996; Engel 2011; Kahneman et al. 1986). This is an economic game where one player has the possibility to donate a certain number of given resources (money or valuable coins) to a second player. The second player can only accept the offer. This game is therefore interpreted to reflect pure giving behavior without strategic reciprocity motivations. As an important building block for social relationships, developmental studies have demonstrated that giving becomes more sensitive to the target (Güroğlu et al. 2014). Whereas giving to unknown others remains relatively stable across adolescence, giving to friends or family increases with age (Güroǧlu et al. 2009; Telzer et al. 2010; van de Groep et al. 2022). Increases in adolescents' giving behavior extend to those in need. In a longitudinal study, Spaans et al. (2023) demonstrated that charity donations increased linearly with age. Moreover, a recent COVID‐19 study showed that, even though adolescents displayed higher giving behavior to societal COVID‐19‐related targets in need (i.e., medical doctors, COVID‐19 patients, and individuals with a poor immune system), giving to societal targets in need decreased with age (Sweijen et al. 2022). In the current study, building upon these previous studies using societal targets in experimental Dictator Games, we examined adolescents' giving behavior to charities as a form of prosocial behavior directed at distant, abstract targets that are generally viewed as legitimate and morally deserving. Unlike close peers, charities represent collective recipients with whom adolescents have no personal relationship and from whom no reciprocity is expected. Charities are often associated with need, fairness, and moral responsibility, which may elicit helping behavior on the basis of internal values rather than relational obligations or social rewards. Although other distant targets (e.g., unknown peers or outgroup members) may engage fairness‐based prosociality, charitable giving more strongly reflects value‐based and altruistically motivated behavior, which makes it a theoretically meaningful contrast to peer‐directed prosocial behavior during adolescence.

A final factor that may be considered a more trait‐like type of prosocial behavior is the social value orientation (Murphy et al. 2011). Social value orientation allows for categorizing individuals into either pro‐selfs (i.e., concerned with maximizing outcomes for self) or pro‐socials (i.e., concerned with maximizing joint outcomes), or a continuous approach to determine the degree of individuals' social preference (Murphy and Ackermann 2014). This social preference can be seen as a personal motive underlying prosocial decisions (van Lange et al. 1998, 2007). Although this may suggest that social value orientation is a personality trait remaining stable across time (Bogaert et al. 2008), whether social value orientation develops across adolescence remains an empirical question yet to be tested longitudinally. In the current study, we investigated the developmental trajectory of adolescents' social preferences.

## 2 The Interplay Between Prosocial and Risk‐Taking Behavior During Adolescence

Although adolescence is known as a period during which prosocial behavior changes, this period in life is also marked by increased risk‐taking. Although there are differences between contexts and between individuals (Blankenstein et al. 2024; Braams et al. 2015; Peper et al. 2018), research suggests a general increase and peak in explorative and risky behavior such as rebelliousness (e.g., alcohol use) across adolescence (Casey 2015; Steinberg 2008). Even though prosocial and risk‐taking behaviors may seem contradictory, recent work on “prosocial risk takers” suggests that these two types of behaviors do not develop in isolation, as adolescents may take risks to benefit others (Do et al. 2017). The same socio‐affective mechanisms and their underlying neural circuitries may partly account for the overlap between prosocial and risk‐taking behavior (Telzer et al. 2013), such that these behaviors are both driven by adolescents' desire for social connection, sensation seeking, and reward drive (e.g., Armstrong‐Carter and Telzer 2024; Carlo et al. 2012). Indeed, adolescents' prosocial actions toward friends and peers are cross‐sectionally associated with rebelliousness, with this combined profile being predicted by reward drive as one underlying factor (Blankenstein et al. 2020). Also, work by Armstrong‐Carter et al. (2022) showed that adolescents show more prosocial behavior on days they also take more risks, which suggests the daily co‐occurrence of prosocial and risk‐taking behavior. Although these initial studies shed light on the temporal association between the two behaviors, it remains unknown how the development of rebelliousness, as one type of risk‐taking (Steinberg 2007, 2008), across adolescence is associated with the development of different types of prosocial behavior. In the current study, we examined adolescents' frequency of engaging in rebellious behavior, such as staying out late and smoking, which is argued to reflect individual's real‐life tendency to take risks (Gullone et al. 2000).

## 3 Age‐ and Puberty‐Related Development

An important question concerns which developmental processes drive changes in prosocial behaviors. Although most prior work examined developmental trajectories of prosocial and risk‐taking behaviors on the basis of chronological age, recent work also suggests differences in these behaviors across pubertal predictors (Ahmed et al. 2020). The onset of puberty, which marks the biological beginning of adolescence, is characterized by rapid hormonal changes and physical maturation (Blakemore et al. 2010). Although chronological age captures how time passes, pubertal status reflects individual variability in biological development. This distinction is critical, as puberty may directly affect adolescents' affective, motivational, and social functioning (Crone and Dahl 2012). In addition, pubertal maturation can shape how adolescents are perceived and treated by others (e.g., peers and adults), which, in turn, may affect behavioral tendencies such as prosocial and risk‐taking behavior (Waylen and Wolke 2004). Therefore, examining both age‐ and puberty‐specific processes provides a more nuanced understanding of developmental changes in adolescents' prosocial and risk‐taking behaviors. Although physical pubertal maturation plays a role in adolescent risk‐taking (e.g., Wierenga et al. 2018), the link to prosocial behaviors remains relatively understudied. The few studies examining the role of physical maturation on prosociality provide evidence that pubertal development drives changes in prosocial behavior above and beyond age. For example, Carlo et al. (2012) demonstrated that early matured adolescents, particularly boys, showed more prosocial behaviors, and work by Ahmed et al. (2020) found puberty effects in prosocial influences independent of age.

Pubertal maturation may be another indicator driving the development of prosocial and risk‐taking behavior. Indeed, prior work has demonstrated that changes in sex steroid hormones (e.g., testosterone and estradiol) are associated with increased risk‐taking, such that sex hormones accelerate the maturational process of risk‐taking during adolescence (Peper and Dahl 2013; Peper et al. 2018). It has been suggested that this effect mainly works through the contribution of hormones on changes in sensation seeking and reward sensitivity. Hormone levels may similarly affect prosocial behavior. Although one study using a prosocial decision‐making task showed that high testosterone was linked with greater prosocial conformity during adolescence (Duell et al. 2021), the link between hormones and prosocial behavior has not yet been investigated in a longitudinal study. We therefore also examined the role of puberty in the development of prosocial behavior and its association with risk‐taking behavior. To best capture multiple aspects of pubertal development (Shirtcliff et al. 2009), both physical (i.e., pubertal development on the basis of secondary sexual characteristics) and hormonal measures (i.e., sex steroids testosterone and estradiol) were used.

## 4 The Current Study

Taken together, given the multidimensionality of prosocial behavior, specific parameters such as type and recipient of prosocial behavior should be taken into account when discussing the developmental trajectories of prosocial behavior (Eisenberg et al. 2006; Fabes et al. 1999; Padilla‐Walker and Carlo 2014). Motivated by prior research, we selected four measures, each tapping into specific prosocial behaviors to examine their developmental trajectories. First, we examined adolescents' prosocial actions to friends and peers, using the self‐report measure Opportunities for Prosocial Actions scale (OPA) in which participants indicate the frequency of performing a variety of prosocial behaviors to their friends and peers (Blankenstein et al. 2020; te Brinke et al. 2023; van de Groep, Zanolie, Green, et al. 2020). Specifically, we examined adolescents' altruism, emotional support, helping, and giving/sharing to friends and peers. Second, we examined adolescents' prosocial tendencies in different situations using the Prosocial Tendencies Measure Revised (PTM‐R) (Carlo et al. 2003). Specifically, we focused on adolescents' public, anonymous, dire, emotional, compliant, and altruistic prosocial behaviors. Third, we studied giving behavior to charities, using the experimental Dictator Game in which participants divided valuable goods between themselves and another charity (target) (Eckel and Grossman 1996; Engel 2011; Kahneman et al. 1986). Fourth, and finally, we studied adolescents' social preference using the Social Value Orientation Slider (SVO) (Murphy et al. 2011) as personal motivation potentially driving prosocial behaviors (van Lange et al. 1998, 2007). We also examined how the development of multidimensional prosocial behavior relates to the development of risk‐taking behavior during adolescence, using the rebelliousness subscale from the self‐reported Adolescent Risk‐Taking Questionnaire (ART) (Gullone et al. 2000).

Our study aims and analysis plan were preregistered and can be found on the Open Science Framework (OSF; https://osf.io/p32jq/). We used longitudinal data from an accelerated cohort study consisting of three assessments for each participant spanning a 5‐year period called ‘Brainlinks’ (Crone et al. 2022). The intervals of approximately 1–2 years between assessments are recommended by a prior study, such that longer time scales (i.e., 2 years) are needed to address changes in prosocial behavior across adolescence (te Brinke et al. 2023). This design allows for a comprehensive examination of adolescent development while capturing both between‐ and within‐person differences. Our first study aim was to describe the developmental trajectories of four types of prosocial behavior (i.e., OPA, PTM‐R, CDG, and SVO) and risk‐taking behavior (i.e., ART) within individuals, and, additionally, how physical and hormonal pubertal development contributes to these developmental trajectories. Here, we also tested gender differences in the development of prosocial and risk‐taking behavior, given that prior studies demonstrated developmental differences between males and females (e.g., Mahalik et al. 2013; Shulman et al. 2015; Van der Graaff et al. 2017). Our second aim was to describe how the trajectories of prosocial behaviors over time are associated with the trajectory of risk‐taking over time during adolescence. Because the COVID‐19 pandemic hit during the period of the three‐wave project this study is part of, we also explored whether the perceived impact of COVID‐19 on adolescents' social relationships and mental health affected the trajectories of prosocial and risk‐taking behavior.

## 5 Method

### 5.1 Participants

This study used data from an accelerated longitudinal three‐wave study called ‘Brainlinks’. Participants between the ages of 9 and 22 were recruited through local and online advertisements. Participants had normal or corrected‐to‐normal vision and no diagnosed intellectual disability (IQ < 70). Participants were screened for (history of) neurological or psychiatric disorders and MRI contraindications (for the MRI scan that was part of the larger study protocol). The study was approved by the METC Leiden‐Den Haag‐Delft and the Psychology Research Ethics committee of Universiteit Leiden. At each timepoint (T1, May–October 2018; T2, August 2019–January 2020; T3, October 2021–June 2022), written informed consent was provided by the participant and, in case of minors, also by their parent(s).

The full sample consisted of 142 participants at T1 (*M*_age_ = 14.46, SD_age_ = 2.77, 90 females, 52 males), 127 participants at T2 (*M*_age_ = 15.81, SD_age_ = 2.69, 79 females, 48 males), and 118 participants at T3 (*M*_age_ = 18.04, SD_age_ = 2.72, 74 females, 44 males). Table 1 presents the demographic information of the samples at each timepoint. Most participants (*n* = 131, 92.3%) were born in the Netherlands, whereas two participants (1.4%) were born in another European country and one (0.7%) in a non‐European country. Participants' socioeconomic status (SES) as measured by parental educational level was middle to high.

**TABLE 1:** Sample characteristics.

	T1	T2	T3
	*N* (% of sample)	*N* (% of sample)	*N* (% of sample)
*N*	142	127	118
Gender			
Female	89 (62.7%)	79 (55.6%)	74 (52.1%)
Male	53 (37.3%)	48 (33.8%)	44 (31.0%)
Education			
Elementary school	34 (23.9%)	14 (9.9%)	0 (0.0%)
High school	88 (62%)	74 (52.1%)	51 (35.9%)
Higher education	12 (8.5%)	29 (20.4%)	59 (41.5%)
Currently no education	0 (0.0%)	9 (6.3%)	5 (3.5%)
Neurological/psychiatric disorder^a^			
Yes	5 (3.5%)	7 (4.9%)	15 (10.6%)
No	124 (87.3%)	117 (82.4%)	98 (69.0%)
I don't know	4 (2.8%)	2 (1.4%)	2 (1.4%)

*Note:* Percentages do not necessarily add up to 100 because of missing data.

^a^ Participants were initially screened for (history of) neurological or psychiatric disorders, but some participants indicated to have neurological and/or psychological disorders during the period of the study. Given that we are interested in the longitudinal trajectories, we decided to not exclude these participants from our analyses.

### 5.2 Procedure

At each timepoint, participants received information about the study through telephone and mail. All original participants from T1 were contacted to participate at the subsequent timepoints if they gave consent to be contacted for a follow‐up study. The intervals between timepoints were approximately 1 year between T1 and T2 (*M* = 1.28 and SD = 0.09), and 2 years between T2 and T3 (*M* = 2.25 and SD = 0.15). The longer assessment interval between T2 and T3 was due to the start of the COVID‐19 pandemic in March 2020. To this end, we aimed to invite participants for T2 and T3 in the same order as they were invited for T1. At each timepoint, the study consisted of three parts. In the first part, participants received questionnaires to be filled in at home prior to the lab visit (including ART, OPA, PTM‐R, and SVO). Second, participants were asked to collect two morning saliva samples at home (for collecting testosterone and estradiol levels), one on the day of the lab visit and another on the day prior to the lab visit. Third, during the lab visit (approximate duration of 3 h), participants underwent an MRI scan (that was part of the larger study protocol), filled in several questionnaires (including PDS), and performed experimental tasks (including CDG). Participants received compensation for their participation (at T1 €20 for ages < 12 years or €30 for ages ≥ 12 years; at T2 €30 for ages < 12 years or €40 for ages ≥ 12 years; at T3 €50 for all ages), as well as small presents and additional earnings from tasks as part of the larger study protocol.

### 5.3 Materials

#### 5.3.1 Prosocial Measures

##### 5.3.1.1 Opportunities for Prosocial Actions (OPA)

The frequency of prosocial actions to friends and peers was assessed with the OPA (Blankenstein et al. 2020; te Brinke et al. 2023; van de Groep, Zanolie, Green, et al. 2020). Covering a broad range of prosocial actions, the scale includes the following four subscales: altruism (10 items, e.g., “Asked if you could help a friend,” Cronbach's *α* at T1 = 0.86), emotional support (8 items, e.g., “Complimented a friend,” Cronbach's *α* at T1 = 0.80), helping solve problems (3 items, e.g., “Helped a friend find a solution,” Cronbach's *α* at T1 = 0.68), and giving/sharing (7 items, e.g., “Gave some money to a friend,” Cronbach's *α* at T1 = 0.80). Participants were asked to indicate how often they displayed these behaviors within the last (few) month(s) using a 6‐point Likert‐scale from 1 (“not something I do”) to 6 (“very often”). Participants received no specific instructions on whether these prosocial actions could be performed online or offline.

##### 5.3.1.2 Prosocial Tendencies Measure—Revised (PTM‐R)

The tendency to show prosocial behaviors in different situations was measured using the PTM‐R (Carlo et al. 2003). The PTM‐R consists of 25 items assessing six types of prosocial behaviors: public (4 items, e.g., “I can help others best when people are watching me,” Cronbach's *α* at T1 = 0.74), anonymous (5 items, e.g., “I prefer to donate money without anyone knowing,” Cronbach's *α* at T1 = 0.74), dire (3 items, e.g., “I tend to help people who are hurt badly,” Cronbach's *α* at T1 = 0.62), emotional (5 items, e.g., “I tend to help others especially when they are really emotional,” Cronbach's *α* at T1 = 0.74), compliant (2 items, e.g., “I never wait to help others when they ask for it,” Cronbach's *α* at T1 = 0.68), and altruism (6 items, e.g., “I often help even if I don't think I will get anything out of helping,” Cronbach's *α* at T1 = 0.73). Participants indicated the extent to which each item applied to them on a scale from 1 (“does not describe me at all”) to 5 (“describes me very well”).

##### 5.3.1.3 Charity Dictator Game (CDG)

Giving behavior to charities was measured using a modified version of the Dictator Game (Eckel and Grossman 1996; Engel 2011; Kahneman et al. 1986). In 10 one‐shot Dictator Games in randomized order, participants divided 10 coins between themselves and different charities (e.g., Amnesty International, Greenpeace, and Unicef). Participants were explained that the coins were worth actual money (i.e., each coin equals 10 eurocents). Participants were also explained that how they divided the coins influenced how much money they and the charities received. We transferred the money on the basis of the actual choices to the participants at the end of the lab visit and to the charities at the end of data collection at each timepoint. At each timepoint, we used the mean score of donated coins across the 10 trials (Cronbach's *α* at T1 = 0.92).

##### 5.3.1.4 Social Value Orientation (SVO)

Social preference was measured with the SVO Slider Measure that examines the intrinsic preference for the distribution of resources (Murphy et al. 2011). For six primary and nine secondary items with a continuum of joint payoffs, participants divided points between themselves and another hypothetical individual by selecting one out of nine fixed choice payoffs (e.g., 90–100, 95–95, and 100–90 for both individuals). Participants played anonymously and were instructed that the points were as valuable to them as to the other individual. Continuous SVO values on the basis of the six primary items were computed as indicated by the angle on a self/other plane, with higher values reflecting greater concern for others' outcomes relative to concern for their own outcomes.

#### 5.3.2 Risk‐Taking Measure

##### 5.3.2.1 Adolescent Risk‐Taking Questionnaire (ART)

Risk‐taking behavior was assessed with the rebellious subscale of the ART (Gullone et al. 2000). This subscale measures the frequency with which participants display risk behaviors (5 items, e.g., “getting drunk” and “staying out late”). Participants were asked whether they experienced the activities on a scale ranging from 0 (“never”) to 4 (“very often”). Mean scores for the subscale at each timepoint were used (Cronbach's *α* at T1 = 0.92). Prior studies across wide age ranges (i.e., 11–28) have shown high reliability and strong test–retest stability (Blankenstein et al. 2018, 2020).

#### 5.3.3 Predictors of Developmental Trajectories

##### 5.3.3.1 Puberty Development Scale (PDS)

Pubertal development was assessed with the PDS (Carskadon and Acebo 1993). This self‐report questionnaire on secondary sexual characteristics (e.g., growth spurt and body hair) uses a 4‐point scale from 1 (“not yet started”) to 4 (“seems complete”). For males, puberty category scores were calculated on the basis of the sum scores of the three individual items on body hair growth, voice change, and facial hair growth (range = 3–12; Cronbach's *α* at T1 = 0.89), with lower sum scores representing less pubertal maturation. For females, category scores were based on the sum of the three items on body hair growth and breast development, while differentiating between females with and without menarche (range = 2–8; Cronbach's *α* at T1 = 0.84). The category scores were pre‐pubertal, early pubertal, mid‐pubertal, late pubertal, and post‐pubertal. Age descriptives per pubertal category from the first timepoint can be found in Table 2.

**TABLE 2:** Age descriptives separated by pubertal development category.

	Females		Males	
	*N*	Mean (range)	*N*	Mean (range)
Pre‐pubertal	0	—	10	11.42 (9.00–13.14)
Early pubertal	0	—	5	11.94 (9.58–13.42)
Mid‐pubertal	0	—	10	15.02 (13.49–18.22)
Late pubertal	45	13.76 (9.16–17.29)	21	16.38 (12.21–18.99)
Post‐pubertal	32	16.86 (14.27–18.95)	4	17.53 (16.68–18.64)

*Note:* As measured by the Pubertal Development Scale (PDS). Sample sizes per category do not necessarily add up to the total sample size (89 females and 53 males) because of missing data.

##### 5.3.3.2 Sex Steroids

Hormonal development was measured using the sex steroids testosterone and estradiol, which were assessed by saliva samples by passive drool. Participants were asked to collect two morning saliva samples at home, one on the day of the lab visit and another on the day prior to the lab visit. Participants were instructed to collect the samples directly after waking up and not to eat or brush their teeth before collecting saliva. In addition, they were asked to store the samples in a freezer at −18°C before the lab visit. The saliva samples were assayed for testosterone and estradiol at the Technical University of Dresden in Germany. Hormone levels were determined by high performance liquid chromatography–tandem mass spectrometry (LC–MS/MS; Gao et al. 2015). At each timepoint, average levels were calculated for the 2 days on which the samples were collected. In case of missing data on 1 day, the value of the single assessment was used. A log transformation was performed on both testosterone and estradiol values at all timepoints because these values showed non‐normal distributions.

##### 5.3.3.3 Perceived Impact of COVID‐19

The perceived impact of COVID‐19 was measured using a seven‐item questionnaire that was developed specifically for and added to T3 (October 2021–June 2022), following the start of the COVID‐19 pandemic in the Netherlands (March 2020). Using a scale ranging from 1 (“totally disagree”) to 7 (“totally agree”), participants indicated the extent to which they had experienced the COVID‐19 pandemic as affecting their social relationships (e.g., with family members, friends, and peers) (6 items) and their mental wellbeing (1 item). An example item is “The COVID‐19 pandemic influenced my relationships with friends.” An average score of the seven items was used (Cronbach's *α* = 0.81; *M* [SD] = 4.41 [1.35]).

### 5.4 Statistical Analyses

The statistical analyses were preregistered on the Open Science Framework (see https://osf.io/p32jq). Deviations from the preregistration are described in Supporting Information S1. To describe the developmental trajectories of prosocial behaviors and risk‐taking behavior (study aim 1), we performed linear mixed models on each measure of prosocial behavior (i.e., subscales of OPA, subscales of PTM‐R, CDG, and SVO) and risk‐taking behavior (i.e., ART), with age and/or puberty, which resulted in 39 models (i.e., 3 models × 13 measures). Quadratic age effects were also examined and, in case of significant effects, used in the models instead of linear age. Subsequently, we added testosterone and estradiol as predictors to the models with both age and puberty to test whether these hormonal measures provide additional variance beyond age and puberty for males and females separately. As such, this methodological approach, which has been used in prior developmental studies (Ahmed et al. 2020; Peper et al. 2018) allowed us to examine the extent to which each pubertal maturation measure is dissociable from chronological age in explaining developmental change. This resulted in 26 additional models (i.e., 2 models × 13 measures). To describe how the development of prosocial behaviors is associated with the development of risk‐taking behavior during adolescence (study aim 2), we first performed univariate Latent Growth Curve (LGC) models on each measure to test for null, linear, and non‐linear trajectories of each prosocial behavior and risk‐taking behavior. Using the models best describing the trajectories over time, we subsequently ran four bivariate LGC models between each prosocial behavior and risk‐taking. To account for multiple testing, we used a Bonferroni correction for correlated variables (Sankoh et al. 1997; Uitenbroek 1997). This resulted in a corrected alpha of 0.006 for study aim 1 and a corrected alpha of 0.015 for study aim 2.

Linear mixed models were performed in R using the lme4 package (Bates et al. 2015; R Core Team 2023), and LGC models were performed in Mplus (version 8.10; Muthén and Muthén 2017). Multiple indices were used to assess the model fit (Hooper et al. 2008; Hu and Bentler 1999; Schermelleh‐Engel et al. 2003): *χ* ^2^ (*p* > 0.05), RMSEA (< 0.08), CFI (> 0.90), Akaike Information Criteria (AIC), and Bayesian Information Criterion (BIC). Outliers, defined by values greater than three box‐lengths from the edge of boxplots, were winsorized (SVO at T1: *n* = 4, SVO at T2: *n* = 2). Post hoc power analyses using the simr package in R (Green and MacLeod 2016) demonstrated sufficient power to detect developmental effects using linear mixed models (e.g., model with linear age on CDG: power = 81%, effect size = 0.12, alpha = 0.05). Regarding the LGC models, model fit convergence was ensured, and key determinants of statistical power, such as confidence intervals (Brandmaier et al. 2018; Schermelleh‐Engel et al. 2003), demonstrated sufficient power to detect the hypothesized slope‐slope associations (e.g., bivariate LGC model between OPA and ART, and rebelliousness: 95% CI [0.003, 0.072], SE = 0.02, *Z* = 2.14). Little's MCAR test indicated that the data (i.e., all longitudinal measures and predictors) were missing completely at random (*χ* ^2^ (2085) = 2189.78, *p* = 0.054), which justifies the use of Full Information Maximum Likelihood (FIML) to handle missing data. That is, we applied the Restricted Maximum Likelihood (REML) in the linear mixed models and the Maximum Likelihood with Robust standard errors (MLR) estimator in the LGC models, which uses maximum likelihood on the basis of the maximum available data per participant. In addition, we examined whether there were baseline differences between participants with and without complete data (i.e., data at each timepoint). Attrition analyses indicated that there were no such significant differences in gender, *χ* ^2^ (1) = 0.36, *p* = 0.548, age, *F* (1,140) = 0.37, *p* = 0.543, and all prosocial and risk‐taking measures (*p* > 0.05). We did not control for gender in our analyses because linear mixed models on each longitudinal measure (i.e., ART, CDG, OPA, PTM‐R, and SVO) indicated that there were no gender differences in developmental trajectories (all interaction effects between gender and age/puberty: *p*'s > 0.05; see Table S1). Sensitivity checks reported in Table S1 showed that the conclusions remained the same when controlling for gender.

## 6 Results

Descriptive statistics and intraclass correlation coefficients (ICCs) are displayed in Table 3. The cross‐sectional correlation matrix among the main variables can be found in Table 4a–c, and the correlations across timepoints and predictors of developmental trajectories (age, puberty, and COVID‐19) are displayed in Table S2.

**TABLE 3:** Descriptive statistics and ICCs for all longitudinal measures and predictors.

Measure	ICC [95% CI]	T1		T2		T3	
		*N*	*M* (SD)	*N*	*M* (SD)	*N*	*M* (SD)
OPA	0.773 [0.681 0.842]	133	3.96 (0.82)	124	3.61 (0.86)	114	3.93 (0.86)
Altruism	0.690 [0.565 0.784]		3.38 (1.02)		2.81 (1.04)		3.19 (1.08)
Emotional support	0.821 [0.748 0.875]		4.14 (1.00)		3.94 (1.11)		4.17 (1.06)
Helping solve problems	0.673 [0.541 0.772]		4.97 (0.81)		4.63 (0.99)		4.88 (0.81)
Giving/sharing	0.699 [0.577 0.790]		3.35 (1.02)		3.07 (1.00)		3.49 (1.04)
PTM‐R	0.681 [0.552 0.777]	133	3.12 (0.41)	120	3.12 (0.48)	117	3.12 (0.43)
Public	0.575 [0.404 0.703]		1.92 (0.76)		1.66 (0.67)		1.74 (0.63)
Anonymous	0.713 [0.597 0.799]		1.96 (0.75)		2.15 (0.89)		2.21 (0.81)
Dire	0.627 [0.477 0.739]		3.71 (0.74)		3.71 (0.84)		3.68 (0.80)
Emotional	0.684 [0.557 0.779]		3.29 (0.76)		3.36 (0.93)		3.20 (0.83)
Compliant	0.467 [0.253 0.628]		3.70 (0.83)		3.66 (0.89)		3.73 (0.86)
Altruism	0.694 [0.570 0.786]		4.13 (0.71)		4.18 (0.60)		4.19 (0.60)
CDG	0.751 [0.658 0.822]	142	5.45 (1.97)	127	6.11 (2.29)	118	6.33 (2.50)
SVO	0.598 [0.431 0.722]	126	32.83 (10.20)	120	33.51 (11.45)	115	31.72 (10.72)
ART	0.910 [0.870 0.939]	110	0.89 (1.09)	121	1.16 (1.17)	111	1.58 (1.28)
Pubertal development							
Females		77	4.42 (0.50)	77	4.18 (0.97)	73	4.68 (0.47)
Males		50	3.08 (1.29)	48	3.44 (1.09)	44	4.07 (0.87)
Sex steroids: testosterone (log)							
Females		83	0.68 (0.47)	76	1.16 (0.28)	66	0.85 (0.30)
Males		51	1.39 (0.79)	44	0.19 (0.51)	34	1.89 (0.39)
Sex steroids: estradiol (log)							
Females		85	0.32 (0.34)	78	0.24 (0.34)	66	0.59 (0.20)
Males		52	0.19 (0.29)	43	0.14 (0.37)	34	0.53 (0.17)

*Note:* The intraclass correlation coefficients (ICCs) were computed in SPSS. Testosterone and estradiol levels were log transformed.

Abbreviations: ART, Adolescent Risk‐Taking Questionnaire; CDG, Charity Dictator Game; OPA, Opportunities for Prosocial Actions; PTM‐*R*, Prosocial Tendencies Measure—Revised; SVO, Social Value Orientation.

**TABLE 4:** Cross‐sectional correlations.

			1	2	3	4	5	6	7	8	9	10	11	12	13
T1															
(A)	T1	1. CDG	1												
		2. OPA altruism	−0.042	1											
		3. OPA emotional support	0.092	0.626**	1										
		4. OPA helping solve problems	−0.140	0.533**	0.662**	1									
		5. OPA giving/sharing	0.012	0.711**	0.651**	0.528**	1								
		6. PTM‐R public	−0.091	0.129	0.070	−0.106	0.073	1							
		7. PTM‐R anonymous	−0.057	0.183*	0.135	0.032	0.149	0.186*	1						
		8. PTM‐R dire	0.008	0.202*	0.184*	0.191*	0.052	0.143	0.200*	1					
		9. PTM‐R emotional	0.113	0.218*	0.321**	0.197*	0.133	0.293**	0.248**	0.623**	1				
		10. PTM‐R compliant	0.030	0.223*	0.290**	0.355**	0.135	−0.016	0.136	0.511**	0.520**	1			
		11. PTM‐R altruism	0.228**	0.053	0.078	0.189*	0.041	−0.538**	−0.283**	0.051	−0.087	0.167	1		
		12. SVO	0.211*	0.143	0.118	0.178	0.082	−0.162	−0.020	0.142	0.120	0.151	0.243**	1	
		13. ART	0.027	0.026	0.151	0.049	0.152	−0.041	0.057	−0.059	−0.112	−0.010	0.024	−0.086	1
T2															
(B)	T2	1. CDG	1												
		2. OPA altruism	−0.155	1											
		3. OPA emotional support	−0.033	0.622**	1										
		4. OPA helping solve problems	0.009	0.403**	0.531**	1									
		5. OPA giving/sharing	−0.153	0.749**	0.712**	0.502**	1								
		6. PTM‐R public	−0.063	0.137	−0.022	0.014	0.106	1							
		7. PTM‐R anonymous	−0.146	0.220*	0.182*	0.172	0.318**	0.238**	1						
		8. PTM‐R dire	0.104	0.288**	0.419**	0.469**	0.343**	0.071	0.236**	1					
		9. PTM‐R emotional	0.096	0.205*	0.292**	0.394**	0.313**	0.192*	0.321**	0.707**	1				
		10. PTM‐R compliant	0.160	0.111	0.242**	0.277**	0.180	0.031	0.239**	0.544**	0.440**	1			
		11. PTM‐R altruism	0.152	−0.112	0.134	0.018	−0.003	−0.416**	−0.156	0.115	−0.100	0.228*	1		
		12. SVO	0.105	0.068	0.057	0.005	0.004	−0.008	−0.012	0.182*	0.092	0.271**	0.290**	1	
		13. ART	0.051	0.164	0.309**	0.126	0.300**	−0.115	−0.050	0.057	0.090	−0.061	−0.118	−0.194*	1
T3															
(C)	T3	1. CDG	1												
		2. OPA altruism	0.081	1											
		3. OPA emotional support	0.102	0.655**	1										
		4. OPA helping solve problems	0.075	0.552**	0.625**	1									
		5. OPA giving/sharing	0.134	0.785**	0.681**	0.652**	1								
		6. PTM‐R public	−0.061	0.051	0.042	−0.031	−0.023	1							
		7. PTM‐R anonymous	−0.026	0.245**	0.133	0.241*	0.290**	0.052	1						
		8. PTM‐R dire	0.045	0.195*	0.371**	0.412**	0.354**	0.071	0.274**	1					
		9. PTM‐R emotional	0.214*	0.176	0.383**	0.440**	0.244**	0.135	0.121	0.567**	1				
		10. PTM‐R compliant	0.073	0.232*	0.350**	0.364**	0.328**	−0.073	0.164	0.467**	0.389**	1			
		11. PTM‐R altruism	0.249**	0.060	0.184	0.155	0.090	−0.488**	0.024	0.240**	0.164	0.284**	1		
		12. SVO	0.207*	−0.139	−0.043	−0.051	0.028	0.045	0.080	0.011	−0.123	−0.035	0.121	1	
		13. ART	0.109	0.029	0.296**	0.092	0.112	0.076	−0.082	0.162	0.194*	0.143	−0.050	−0.084	1

*Note:* Cross‐sectional correlations among main variables at T1 (A), T2 (B), and T3 (C).

Abbreviations: ART, Adolescent Risk‐Taking Questionnaire; CDG, Charity Dictator Game; OPA, Opportunities for Prosocial Actions; PTM‐*R*, Prosocial Tendencies Measure—Revised; SVO, Social Value Orientation.

* *p* < 0.05.

** *p* < 0.01.

### 6.1 Developmental Trajectories of Prosocial and Risk‐Taking Behavior

**FIGURE 1:**
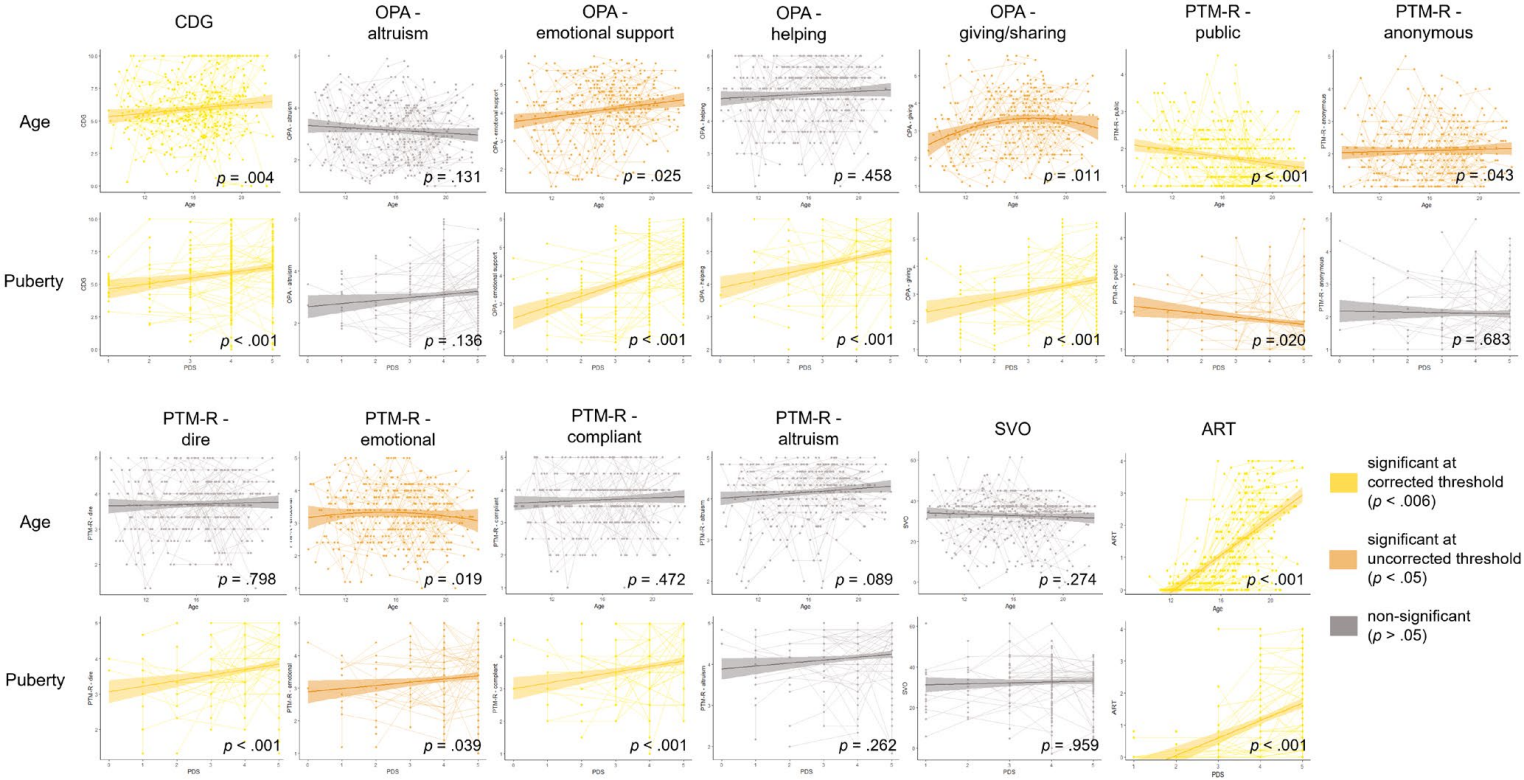
Overview of developmental trajectories on the basis of age (uncorrected for puberty) and puberty (uncorrected for age) of CDG (Charity Dictator Game), subscales of OPA (Opportunities for Prosocial Actions), subscales of PTM‐R (Prosocial Tendencies Measure—Revised), SVO (Social Value Orientation), and ART (Adolescent Risk‐Taking Questionnaire).

We first describe the developmental trajectories of the different types of prosocial behaviors (i.e., subscales of OPA, subscales of PTM‐R, CDG, and SVO) and risk‐taking behavior (i.e., ART). In Figure 1, we present an overview of all developmental effects.

#### 6.1.1 Age‐ and Puberty‐Related Developmental Patterns of Prosocial Behaviors

The results of the linear mixed models are displayed in Table 5, including AIC and BIC values of each model. Here, we first present the results using age and puberty as predictors in the models. We report and interpret the effects that are significant at the corrected threshold.

**TABLE 5:** Linear mixed models including age and pubertal development.

Measure	Model	*β*	SE	ANOVA	AIC	BIC
CDG	Intercept	4.00	0.67		1666.73	1682.57
	Linear age	0.12	0.04	*F* (1) = 8.50, *p* = 0.004		
	Intercept	4.09	0.52		1585.18	1600.82
	PDS	0.44	0.12	*F* (1) = 12.84, *p* < 0.001		
	Intercept	3.38	0.72		1589.12	1608.68
	Linear age	0.08	0.06	*F* (1) = 2.04, *p* = 0.155		
	PDS	0.30	0.16	*F* (1) = 3.67, *p* = 0.056		
OPA—altruism	Intercept	3.64	0.33		1067.94	1083.61
	Linear age	−0.03	0.02	*F* (1) = 2.29, *p* = 0.131		
	Intercept	2.79	0.24		1024.56	1040.07
	PDS	0.09	0.06	*F* (1) = 2.23, *p* = 0.136		
	Intercept	3.37	0.35		1026.47	1045.85
	Linear age	−0.06	0.03	*F* (1) = 5.55, *p* = 0.019		
	PDS	0.19	0.07	*F* (1) = 7.05, *p* = 0.008		
OPA—emotional support	Intercept	3.42	0.04		1001.24	1016.91
	Linear age	0.04	0.02	*F* (1) = 5.09, *p* = 0.025		
	Intercept	2.95	0.22		939.30	954.81
	PDS	0.28	0.05	*F* (1) = 28.65, *p* < 0.001		
	Intercept	2.86	0.32		946.76	966.15
	Linear age	0.01	0.02	*F* (1) = 0.20, *p* = 0.658		
	PDS	0.26	0.06	*F* (1) = 16.16, *p* < 0.001		
OPA—helping solve problems	Intercept	4.64	0.27		935.46	951.12
	Linear age	0.01	0.02	*F* (1) = 0.55, *p* = 0.458		
	Intercept	3.94	0.19		875.11	890.62
	PDS	0.22	0.05	*F* (1) = 22.17, *p* < 0.001		
	Intercept	4.33	0.28		879.14	898.53
	Linear age	−0.04	0.02	*F* (1) = 3.80, *p* = 0.052		
	PDS	0.29	0.06	*F* (1) = 24.16, *p* < 0.001		
OPA—giving/sharing	Intercept	2.95	0.35		1030.17	1049.75
	Linear age	0.03	0.02	*F* (1) = 3.16, *p* = 0.076		
	Quadratic age	−0.03	0.01	*F* (1) = 6.55, *p* = 0.011		
	Intercept	2.52	0.23		977.95	993.46
	PDS	0.20	0.05	*F* (1) = 13.38, *p* < 0.001		
	Intercept	2.78	0.36		989.66	1012.93
	Linear age	0.00	0.02	*F* (1) = 0.02, *p* = 0.880		
	Quadratic age	−0.02	0.01	*F* (1) = 5.04, *p* = 0.026		
	PDS	0.16	0.07	*F* (1) = 5.45, *p* = 0.020		
PTM‐R—public	Intercept	2.51	0.21		761.75	777.40
	Linear age	−0.05	0.01	*F* (1) = 12.85, *p* < 0.001		
	Intercept	2.10	0.15		728.27	743.78
	PDS	−0.08	0.04	*F* (1) = 5.47, *p* = 0.020		
	Intercept	2.44	0.22		731.75	751.14
	Linear age	−0.04	0.02	*F* (1) = 4.74, *p* = 0.030		
	PDS	−0.02	0.05	*F* (1) = 0.11, *p* = 0.744		
PTM‐R—anonymous	Intercept	1.61	0.25		851.26	866.92
	Linear age	0.03	0.02	*F* (1) = 4.12, *p* = 0.043		
	Intercept	2.04	0.18		828.49	844.00
	PDS	0.02	0.04	*F* (1) = 0.17, *p* = 0.683		
	Intercept	1.60	0.26		831.51	850.90
	Linear age	0.05	0.02	*F* (1) = 4.94, *p* = 0.027		
	PDS	−0.06	0.06	*F* (1) = 1.13, *p* = 0.289		
PTM‐R—dire	Intercept	3.64	0.24		855.90	871.56
	Linear age	0.00	0.01	*F* (1) = 0.07, *p* = 0.798		
	Intercept	3.13	0.17		810.70	826.21
	PDS	0.14	0.04	*F* (1) = 11.39, *p* < 0.001		
	Intercept	3.49	0.25		814.73	834.11
	Linear age	−0.04	0.02	*F* (1) = 3.99, *p* = 0.047		
	PDS	0.21	0.05	*F* (1) = 15.16, *p* < 0.001		
PTM‐R—emotional	Intercept	3.74	0.28		888.65	908.21
	Linear age	−0.02	0.02	*F* (1) = 1.49, *p* = 0.223		
	Quadratic age	−0.02	0.01	*F* (1) = 5.66, *p* = 0.019		
	Intercept	3.02	0.19		852.60	868.11
	PDS	0.07	0.04	*F* (1) = 2.19, *p* = 0.140		
	Intercept	3.66	0.29		861.71	884.98
	Linear age	−0.05	0.02	*F* (1) = 4.58, *p* = 0.033		
	Quadratic age	−0.02	0.01	*F* (1) = 4.39, *p* = 0.038		
	PDS	0.12	0.06	*F* (1) = 4.31, *p* = 0.039		
PTM‐R—compliant	Intercept	3.51	0.26		932.83	948.49
	Linear age	0.01	0.02	*F* (1) = 0.52, *p* = 0.472		
	Intercept	3.07	0.19		886.95	902.46
	PDS	0.15	0.04	*F* (1) = 11.85, *p* < 0.001		
	Intercept	3.41	0.27		891.57	910.96
	Linear age	−0.04	0.02	*F* (1) = 3.23, *p* = 0.073		
	PDS	0.23	0.06	*F* (1) = 14.47, *p* < 0.001		
PTM‐R—altruism	Intercept	3.84	0.19		664.03	679.68
	Linear age	0.02	0.01	*F* (1) = 2.91, *p* = 0.089		
	Intercept	4.02	0.14		630.45	645.96
	PDS	0.04	0.03	*F* (1) = 1.26, *p* = 0.262		
	Intercept	3.89	0.20		638.02	657.41
	Linear age	0.02	0.02	*F* (1) = 0.91, *p* = 0.341		
	PDS	0.01	0.04	*F* (1) = 0.06, *p* = 0.802		
SVO	Intercept	36.25	3.35		2715.03	2730.59
	Linear age	−0.22	0.21	*F* (1) = 1.20, *p* = 0.274		
	Intercept	32.41	2.61		2593.18	2608.55
	PDS	0.03	0.62	*F* (1) = 0.00, *p* = 0.959		
	Intercept	35.93	3.63		2593.88	2613.09
	Linear age	−0.40	0.29	*F* (1) = 1.93, *p* = 0.165		
	PDS	0.78	0.81	*F* (1) = 0.91, *p* = 0.341		
ART	Intercept	−3.21	0.30		822.24	837.58
	Linear age	0.27	0.02	*F* (1) = 221.77, *p* < 0.001		
	Intercept	−0.65	0.26		938.74	954.05
	PDS	0.44	0.06	*F* (1) = 51.62, *p* < 0.001		
	Intercept	−3.23	0.30		821.96	841.09
	Linear age	0.28	0.02	*F* (1) = 148.58, *p* < 0.001		
	PDS	−0.06	0.06	*F* (1) = 0.81, *p* = 0.369		

Abbreviations: AIC, Akaike Information Criterion; ART, Adolescent Risk‐Taking Questionnaire; BIC, Bayesian Information Criterion; CDG, Charity Dictator Game; OPA, Opportunities for Prosocial Actions; PDS, Pubertal Development Scale; PTM‐*R*, Prosocial Tendencies Measure—Revised; SVO, Social Value Orientation.

Regarding the different OPA subscales, we found no effects of age (*p* = 0.131) and puberty (*p* = 0.136) on altruism, suggesting age‐related stability of altruism to friends and peers across adolescence. For emotional support, we found no age effects at the corrected threshold (*p* = 0.025). Results showed a puberty‐related increase (*β* = 0.28, *p* < 0.001), which remained significant even when controlling for age (*β* = 0.26, *p* < 0.001), suggesting that emotional support to friends and peers increases with puberty and not with age. For helping solve problems, we found no effects of age (*p* = 0.458). However, we did find a puberty‐related increase (*β* = 0.22, *p* < 0.001), which remained significant when controlling for age (*β* = 0.29, *p* < 0.001), suggesting that helping friends and peers increases with puberty and not with age. For giving/sharing, we found no age‐related effects at the corrected threshold (*p* = 0.011). However, we found a puberty‐related increase (*β* = 0.20, *p* < 0.001), suggesting that giving and sharing behavior to friends and peers increases with puberty.

Regarding the different PTM‐R subscales, results demonstrated an age‐related decrease in public prosocial behavior (*β* = −0.05, *p* < 0.001), suggesting that prosocial behavior in a public setting decreases with age. We found no puberty‐related effects at a corrected threshold (*p* = 0.020). For the anonymous subscale, we detected no effects of age at a corrected threshold (*p* = 0.043) and no effects of puberty (*p* = 0.683), suggesting stability of anonymous prosocial behavior across adolescence. For the dire subscale, we detected no age effects at the corrected threshold (*p* = 0.047). However, results demonstrated a puberty‐related increase (*β* = 0.14, *p* < 0.001), which remained significant when controlling for age (*β* = 0.21, *p* < 0.001), suggesting an increase of dire prosocial behavior specifically associated with pubertal development rather than age. For the emotional subscale, there were no corrected effects of age (*p* = 0.019), as well as no effects of puberty (*p* = 0.140). For the compliant subscale, there were no age effects (*p* = 0.472), suggesting that compliant prosocial behavior remains stable across age. However, we did find a puberty‐related increase (*β* = 0.15, *p* < 0.001), which remained significant when controlling for age (*β* = 0.23, *p* < 0.001), suggesting that compliant prosocial behavior increases with puberty and not with age. For altruism, we found no age‐related changes (*p* = 0.089) as well as no puberty‐related changes (*p* = 0.262). These results suggest that altruistic prosocial behavior remains stable across adolescence.

For CDG, we found age‐ and puberty‐related increases (*β* = 0.12, *p* = 0.004 and *β* = 0.44, *p* < 0.001, respectively), showing that giving behavior to charities increases with age and pubertal development.

Finally, there were no age‐related changes (*p* = 0.274) as well as no puberty‐related changes in SVO (*p* = 0.959), suggesting that concern for others remains stable across adolescence.

#### 6.1.2 Hormonal‐Related Developmental Patterns of Prosocial Behaviors

We subsequently added testosterone and estradiol to the models with age and puberty, separately for males and females, to examine hormone‐related patterns in prosocial behaviors (see Table 6). We found effects of testosterone and estradiol on several subscales of the OPA. For altruism, results demonstrated a testosterone‐related decrease for females (*β* = −0.46, *p* = 0.005), but not for males (*p* = 0.247), suggesting that altruism to friends and peers decreases as testosterone increases among female adolescents. For emotional support, we also detected hormonal effects, such that we found a testosterone‐related decrease among females (*β* = −0.29, *p* = 0.019) and an estradiol‐related increase among males (*β* = 0.62, *p* = 0.043), but these were not significant at a corrected threshold. Similarly, for giving/sharing, results showed a testosterone‐related decrease for females at an uncorrected threshold (*β* = −0.39, *p* = 0.010), but not for males (*p* = 0.617).

**TABLE 6:** Linear mixed models including hormonal measures.

Measure	Model	Females			Males		
		*β*	SE	ANOVA	*β*	SE	ANOVA
CDG	Intercept	4.08	1.04		2.20	1.53	
	Linear age	0.15	0.07	*F* (1) = 4.14, *p* = 0.043	0.12	0.14	*F* (1) = 0.74, *p* = 0.392
	PDS	−0.05	0.25	*F* (1) = 0.04, *p* = 0.851	−0.10	0.32	*F* (1) = 0.09, *p* = 0.769
	Testosterone	0.16	0.31	*F* (1) = 0.27, *p* = 0.607	0.81	0.50	*F* (1) = 2.61, *p* = 0.109
	Estradiol	−0.44	0.40	*F* (1) = 1.18, *p* = 0.280	0.60	0.70	*F* (1) = 0.71, *p* = 0.402
OPA—altruism	Intercept	3.98	0.49		3.30	0.67	
	Linear age	−0.07	0.03	*F* (1) = 4.45, *p* = 0.036	−0.04	0.06	*F* (1) = 0.32, *p* = 0.572
	PDS	0.21	0.11	*F* (1) = 3.94, *p* = 0.049	0.15	0.15	*F* (1) = 0.99, *p* = 0.321
	Testosterone	−0.46	0.16	*F* (1) = 7.98, *p* = 0.005	−0.27	0.23	*F* (1) = 1.35, *p* = 0.247
	Estradiol	0.04	0.20	*F* (1) = 0.04, *p* = 0.839	0.19	0.32	*F* (1) = 0.33, *p* = 0.568
OPA—emotional support	Intercept	3.65	0.43		2.87	0.62	
	Linear age	0.03	0.03	*F* (1) = 0.76, *p* = 0.386	−0.02	0.06	*F* (1) = 0.14, *p* = 0.713
	PDS	0.13	0.08	*F* (1) = 2.43, *p* = 0.121	0.24	0.14	*F* (1) = 2.73, *p* = 0.102
	Testosterone	−0.29	0.12	*F* (1) = 5.61, *p* = 0.019	0.02	0.22	*F* (1) = 0.01, *p* = 0.919
	Estradiol	0.05	0.16	*F* (1) = 0.09, *p* = 0.766	0.62	0.30	*F* (1) = 4.19, *p* = 0.043
OPA—helping solve problems	Intercept	4.18	0.40		5.23	0.57	
	Linear age	−0.04	0.03	*F* (1) = 1.49, *p =* 0.223	−0.09	0.05	*F* (1) = 3.14, *p* = 0.079
	PDS	0.35	0.09	*F* (1) = 15.49, *p* < 0.001	0.28	0.13	*F* (1) = 4.35, *p* = 0.039
	Testosterone	−0.20	0.14	*F* (1) = 2.13, *p* = 0.146	−0.10	0.20	*F* (1) = 0.24, *p* = 0.621
	Estradiol	0.03	0.17	*F* (1) = 0.03, *p* = 0.853	0.38	0.28	*F* (1) = 1.85, *p* = 0.177
OPA—giving/sharing	Intercept	3.11	0.52		2.85	0.66	
	Linear age	0.01	0.03	*F* (1) = 0.17, *p* = 0.680	0.02	0.06	*F* (1) = 0.09, *p* = 0.765
	Quadratic age	−0.01	0.01	*F* (1) = 0.64, *p* = 0.426	−0.039	0.02	*F* (1) = 5.21, *p* = 0.026
	PDS	0.12	0.10	*F* (1) = 1.33, *p* = 0.251	0.12	0.15	*F* (1) = 0.63, *p* = 0.427
	Testosterone	−0.39	0.15	*F* (1) = 6.79, *p* = 0.010	−0.12	0.23	*F* (1) = 0.25, *p* = 0.617
	Estradiol	0.14	0.19	*F* (1) = 0.53, *p* = 0.466	0.00	0.31	*F* (1) = 0.00, *p* = 0.999
PTM‐R—public	Intercept	2.42	0.32		2.63	0.45	
	Linear age	−0.07	0.02	*F* (1) = 7.27, *p* = 0.008	−0.05	0.04	*F* (1) = 1.69, *p* = 0.197
	PDS	0.08	0.08	*F* (1) = 1.04, *p* = 0.308	0.00	0.11	*F* (1) = 0.00, *p* = 0.964
	Testosterone	−0.05	0.11	*F* (1) = 0.22, *p* = 0.640	0.10	0.17	*F* (1) = 0.32, *p* = 0.573
	Estradiol	0.11	0.14	*F* (1) = 0.58, *p* = 0.446	0.01	0.23	*F* (1) = 0.00, *p* = 0.967
PTM‐R—anonymous	Intercept	1.66	0.38		1.01	0.56	
	Linear age	0.00	0.03	*F* (1) = 0.00, *p* = 0.947	0.10	0.05	*F* (1) = 4.12, *p* = 0.045
	PDS	0.06	0.09	*F* (1) = 0.43, *p* = 0.515	−0.13	0.12	*F* (1) = 1.18, *p* = 0.279
	Testosterone	0.04	0.12	*F* (1) = 0.12, *p* = 0.731	−0.03	0.19	*F* (1) = 0.03, *p* = 0.854
	Estradiol	0.26	0.16	*F* (1) = 2.66, *p* = 0.105	−0.15	0.25	*F* (1) = 0.34, *p* = 0.559
PTM‐R—dire	Intercept	3.36	0.39		3.08	0.48	
	Linear age	−0.05	0.03	*F* (1) = 3.52, *p* = 0.062	0.00	0.04	*F* (1) = 0.00, *p* = 0.970
	PDS	0.32	0.09	*F* (1) = 11.35, *p* < 0.001	0.12	0.11	*F* (1) = 1.23, *p* = 0.270
	Testosterone	−0.10	0.13	*F* (1) = 0.54, *p* = 0.465	0.07	0.17	*F* (1) = 0.16, *p* = 0.693
	Estradiol	−0.02	0.16	*F* (1) = 0.01, *p* = 0.904	−0.10	0.23	*F* (1) = 0.21, *p* = 0.649
PTM‐R—emotional	Intercept	3.67	0.47		3.38	0.51	
	Linear age	−0.05	0.03	*F* (1) = 2.39, *p* = 0.124	−0.04	0.04	*F* (1) = 0.85, *p* = 0.358
	Quadratic age	−0.02	0.01	*F* (1) = 3.96, *p* = 0.050	−0.02	0.01	*F* (1) = 1.39, *p* = 0.243
	PDS	0.15	0.10	*F* (1) = 1.91, *p* = 0.168	0.06	0.11	*F* (1) = 0.31, *p* = 0.582
	Testosterone	−0.03	0.14	*F* (1) = 0.06, *p* = 0.809	0.22	0.18	*F* (1) = 1.47, *p* = 0.229
	Estradiol	0.03	0.18	*F* (1) = 0.02, *p* = 0.879	−0.09	0.23	*F* (1) = 0.14, *p* = 0.713
PTM‐R—compliant	Intercept	3.42	0.39		3.43	0.55	
	Linear age	−0.01	0.03	*F* (1) = 0.23, *p* = 0.633	−0.04	0.05	*F* (1) = 0.57, *p* = 0.453
	PDS	0.16	0.10	*F* (1) = 2.39, *p* = 0.124	0.29	0.12	*F* (1) = 5.41, *p* = 0.022
	Testosterone	−0.11	0.14	*F* (1) = 0.62, *p* = 0.431	−0.21	0.20	*F* (1) = 1.09, *p* = 0.300
	Estradiol	0.12	0.18	*F* (1) = 0.43, *p* = 0.511	−0.22	0.27	*F* (1) = 0.65, *p* = 0.423
PTM‐R—altruism	Intercept	3.99	0.27		3.42	0.45	
	Linear age	0.04	0.02	*F* (1) = 3.39, *p* = 0.067	0.03	0.04	*F* (1) = 0.62, *p* = 0.432
	PDS	−0.09	0.06	*F* (1) = 2.02, *p* = 0.157	−0.03	0.10	*F* (1) = 0.10, *p* = 0.754
	Testosterone	0.08	0.08	*F* (1) = 0.96, *p* = 0.329	0.12	0.16	*F* (1) = 0.53, *p* = 0.469
	Estradiol	0.00	0.11	*F* (1) = 0.00, *p* = 0.982	−0.10	0.21	*F* (1) = 0.23, *p* = 0.634
SVO	Intercept	34.42	4.95		37.12	8.42	
	Linear age	0.10	0.36	*F* (1) = 0.08, *p* = 0.781	−0.49	0.75	*F* (1) = 0.41, *p* = 0.525
	PDS	−0.41	1.26	*F* (1) = 0.10, *p* = 0.748	−0.62	1.82	*F* (1) = 0.11, *p* = 0.739
	Testosterone	0.96	1.64	*F* (1) = 0.34, *p* = 0.563	1.60	2.84	*F* (1) = 0.31, *p* = 0.579
	Estradiol	−2.77	2.01	*F* (1) = 1.87, *p* = 0.174	0.64	3.85	*F* (1) = 0.03, *p* = 0.870
ART	Intercept	−3.91	0.48		−3.12	0.57	
	Linear age	0.30	0.03	*F* (1) = 82.26, *p* < 0.001	0.26	0.05	*F* (1) = 29.50, *p* < 0.001
	PDS	−0.01	0.11	*F* (1) = 0.01, *p* = 0.938	0.01	0.11	*F* (1) = 0.01, *p* = 0.921
	Testosterone	0.31	0.14	*F* (1) = 5.10, *p* = 0.025	−0.02	0.18	*F* (1) = 0.02, *p* = 0.892
	Estradiol	−0.42	0.17	*F* (1) = 5.91, *p* = 0.016	0.03	0.24	*F* (1) = 0.01, *p* = 0.904

Abbreviations: ART, Adolescent Risk‐Taking Questionnaire; CDG, Charity Dictator Game; OPA, Opportunities for Prosocial Actions; PDS, Pubertal Development Scale; PTM‐*R*, Prosocial Tendencies Measure—Revised; SVO, Social Value Orientation.

There were no significant hormone‐related effects for giving to charities, helping friends and peers, all subscales of PTM‐R (public, anonymous, dire, emotional, compliant, and altruism), and concern for others (all *p*'s > 0.05), suggesting no hormone‐related changes in these prosocial behaviors.

#### 6.1.3 Age‐, Puberty‐, and Hormonal‐Related Developmental Patterns of Risk‐Taking

For risk‐taking (i.e., ART), we performed the same models on the basis of age and puberty (step 1), and separately for females and males, testosterone and estradiol (step 2). Results demonstrated age effects (*β* = 0.27, *p* < 0.001), also when controlled for puberty (*β* = 0.28, *p* < 0.001), suggesting an age‐related increase in risk‐taking. We also found a puberty effect (*β* = 0.44, *p* < 0.001), but not when controlling for age (*p* = 0.369), suggesting a puberty‐ and age‐related increase in risk‐taking. Finally, we detected hormonal‐related changes among females, such that there was a testosterone‐related increase (*β* = 0.31, *p* = 0.025) and an estradiol‐related decrease for females (*β* = −0.42, *p* = 0.016), but these effects were non‐significant at a corrected threshold. There were no hormonal‐related changes among males (*p* = 0.892 and *p* = 0.904, for testosterone and estradiol respectively).

### 6.2 Associations Between Prosocial and Risk‐Taking Behavior

**FIGURE 2:**
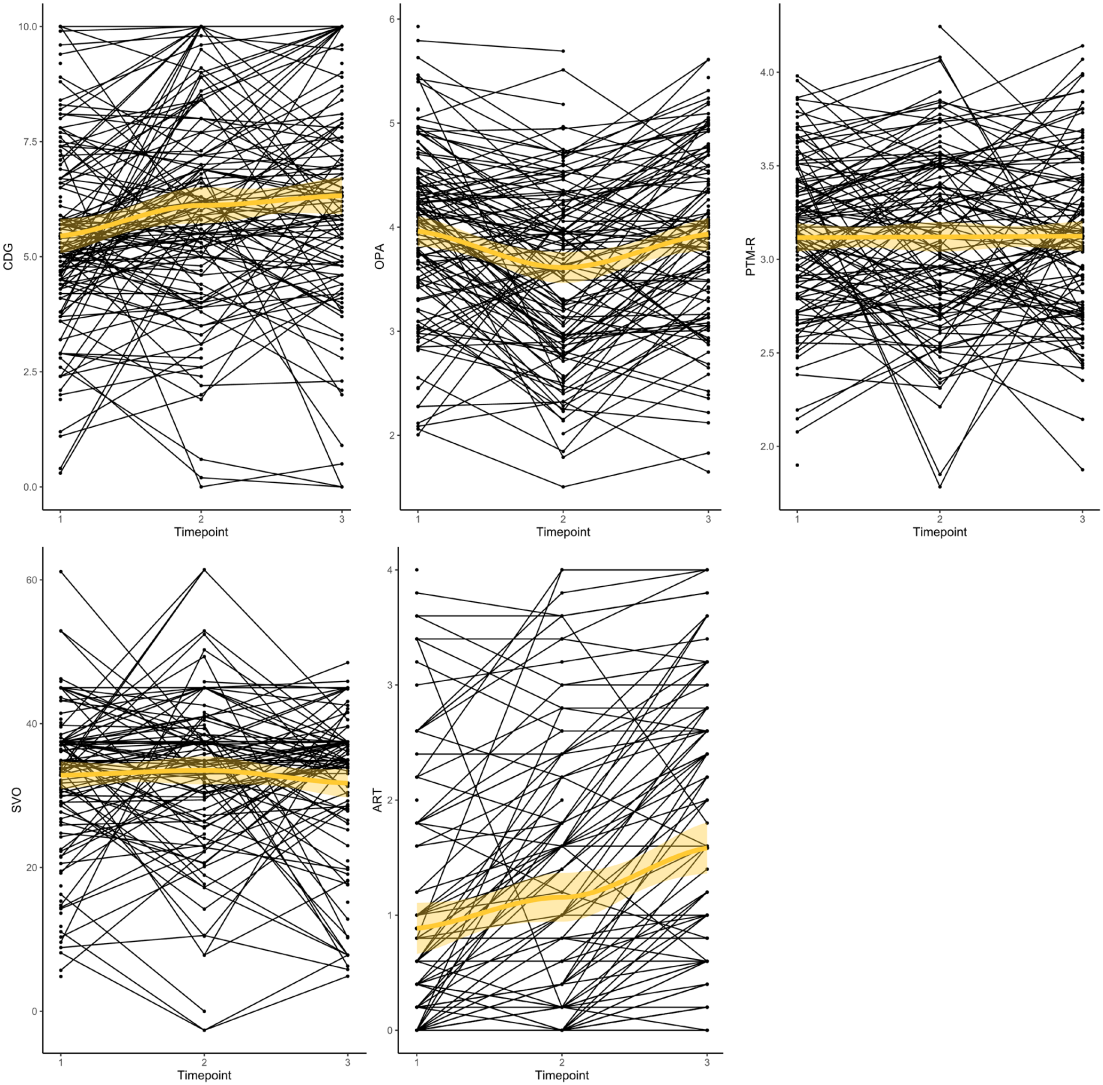
Time effects of raw data on all longitudinal measures. The trend line shows the mean with the outline representing the 95% confidence interval. Although CDG (Charity Dictator Game) was best described by a non‐linear increase over time, OPA (Opportunities for Prosocial Actions) was best described by a non‐linear decrease. PTM‐R (Prosocial Tendencies Measure—Revised) and SVO (Social Value Orientation) showed no changes over time. Risk‐taking (i.e., ART; Adolescent Risk‐Taking Questionnaire) was best described by a linear increase over time.

Our second aim was to describe how the developmental trajectories of the four types of prosocial behaviors are associated with the development of risk‐taking during adolescence. To find the model best describing these trajectories, we first performed univariate LGC models for each prosocial behavior and risk‐taking behavior by testing null (intercept‐only model), linear (including a fixed and random linear slope model), and non‐linear (including a fixed and random non‐linear slope model) models. Model fit indices are displayed in Table S5. Time effects of raw data are shown in Figure 2. Here, we used average scores on the subscales of OPA and PTM‐R as model fit indices showed bad fit in the models on each subscale separately (see Table S4). Results showed that CDG and OPA were best described by non‐linear models with random slopes. Although CDG showed a non‐linear increase over time (*β* = 0.35, *p* = 0.001), OPA showed a non‐linear decrease over time (*β* = −0.93, *p* < 0.001). This indicates that giving behavior to charities *increased* over time, with a less steep increase from T2 to T3, whereas prosocial behavior to peers/friends *decreased* over time, with a steeper decrease from T1 to T2. PTM‐R was best described by an intercept‐only model, thereby indicating that prosocial tendencies did not change over time. Finally, SVO was best described by a linear model with random slopes, but results revealed that the slope (*β* = −0.07) was non‐significant (*p* = 0.512), indicating that adolescents' social preference did not change over time. Regarding risk‐taking (i.e., ART), risk‐taking behavior was best described by a linear model with random slopes (see also Figure 2), as indicated by model fit indices in Table S6. The positive slope of *β* = 0.77 (*p* < 0.001) suggests a linear increase over time. Thus, risk‐taking increases over time.

**FIGURE 3:**
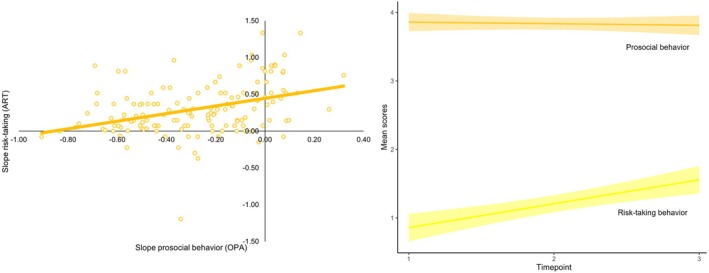
Association between the slope of risk‐taking behavior (ART; Adolescent Risk‐Taking Questionnaire) and the slope of prosocial behavior (OPA; Opportunities for Prosocial Actions).

We then examined whether the development of risk‐taking behavior is associated with the development of prosocial behavior. Specifically, we tested whether the intercept (i.e., average initial level) and slope (i.e., average change over time) of risk‐taking were associated with the intercept and slope of each prosocial behavior by performing bivariate LGC models, using the best‐fitting univariate models from the previous step. Model fit indices are displayed in Table S7. Results revealed that the slope of OPA was positively associated with the slope of risk‐taking behavior (*β* = 0.04, *p* = 0.032, see Figure 3), but this effect was not significant at a corrected threshold. There were no significant covariances between risk‐taking and the other prosocial behaviors (i.e., CDG, PTM‐R, and SVO).

### 6.3 Perceived Impact of COVID‐19 on the Trajectories of Prosocial and Risk‐Taking Behavior

**FIGURE 4:**
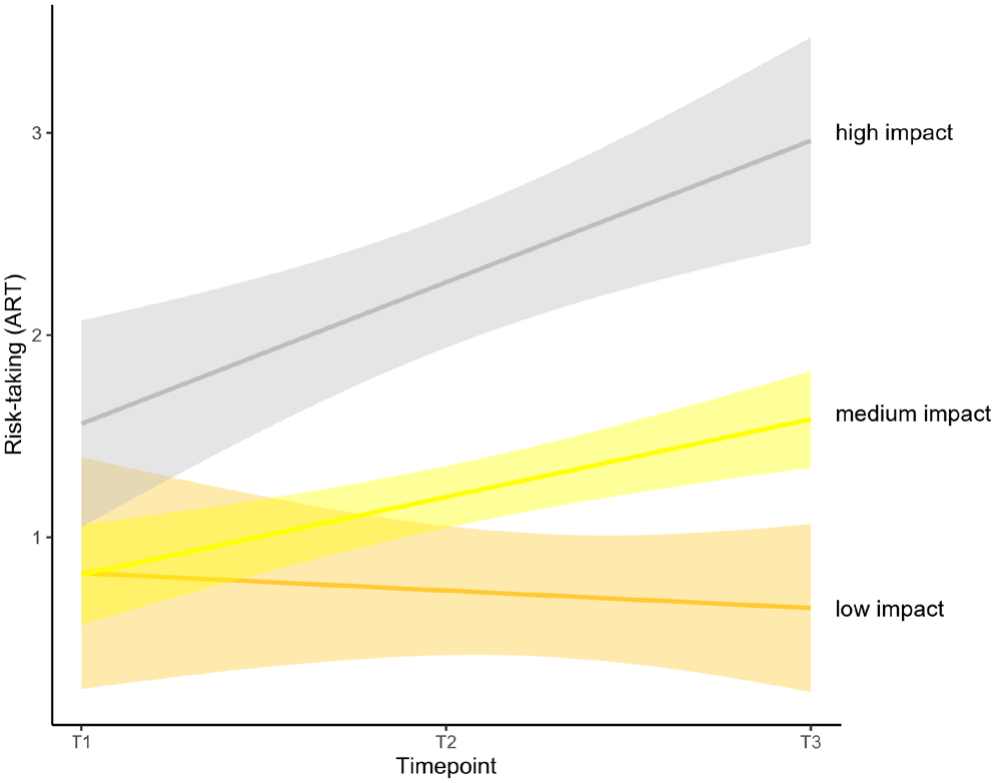
Association between perceived impact of the COVID‐19 pandemic on risk‐taking behavior, specifically rebelliousness (ART; Adolescent Risk‐Taking Questionnaire). For illustrative purposes, categorical groups were created on the basis of whether individuals showed values in perceived impact of COVID‐19 that were lower than 1 SD from the mean (low impact: *n* = 20), no more than 1 SD around the mean (medium impact: *n* = 85), and higher than 1 SD from the mean (high impact: *n* = 12).

Additionally, we explored the extent to which the developments over time of CDG, OPA, and ART were affected by the perceived impact of COVID‐19 by adding the latter as a predictor on the slopes. Results revealed no effect of the perceived impact of COVID‐19 on the development of CDG (*β* = −0.10, *p* = 0.236) and OPA (*β* = 0.22, *p* = 0.184), which indicates that the increase in giving behavior to charities and the decrease in prosocial actions to friends and peers from T2 (i.e., before the pandemic) to T3 (i.e., during the pandemic) were not influenced by the perceived impact of the pandemic. However, we did find an uncorrected positive correlation between the impact of COVID‐19 and OPA at T3 (*r* = 0.20, *p* = 0.037), suggesting that adolescents who indicated that the pandemic impacted their social relationships and mental health more also showed more prosocial behavior to friends and peers during the pandemic. Adding the perceived impact of COVID‐19 as a predictor to the risk‐taking model revealed an effect on the slope (*β* = 0.43, *p* < 0.001). As shown in Figure 4, adolescents who indicated they were impacted more by the COVID‐19 pandemic showed a greater increase in risk‐taking from T2 (i.e., before the pandemic) to T3 (i.e., during the pandemic).

## 7 Discussion

In the current study, we examined the developmental trajectories of multiple types of prosocial behaviors within individuals during adolescence, and their associations with the development of rebelliousness. Specifically, we tested a variety of prosocial actions directed to friends and peers, prosocial behaviors in different situations, giving to charities, and social value orientation to unravel developmental time courses for prosocial behaviors toward close and distant targets. Our study demonstrated that the developmental trajectories of prosocial behaviors depend on the type, target, and situation. By examining age‐, puberty‐, and hormonal‐related trajectories, we discovered that physical pubertal development was a stronger predictor of prosocial behaviors than chronological age. The current study contributes to the larger literature on the multidimensionality of prosocial behavior (Padilla‐Walker and Carlo 2014), showing that this multidimensionality should be considered for understanding the development of prosociality during adolescence.

### 7.1 Developmental Trajectories of Prosocial Behavior

Our study demonstrates that prosocial behavior does not follow one developmental trajectory across adolescence. Although some behaviors (i.e., social value orientation and altruism) remain stable across adolescence, the behaviors that show change mostly show developmental increases, specifically for emotional support, helping to solve problems, giving/sharing toward friends, as well as dire and compliant prosocial motivations, and giving to charity. In contrast, public prosocial behavior showed a developmental decrease. Given that the developmental trajectories depend on the type, situation, and receiver of the prosocial behavior, our results suggest that prosocial behavior shows a highly context‐driven development. An important novel finding of this longitudinal design is that puberty demonstrated a stronger predictor of developmental changes than age. In summary, the following patterns in developmental trajectories can be detected.

First, we found age‐ and puberty‐related increases in adolescents' giving behavior. Specifically, giving and sharing behavior to friends and peers increases with puberty, and giving behavior to charities increases with age and puberty. These results are in line with previous studies demonstrating age‐related increases to in‐group members, such as friends and family but also to those in need (Güroǧlu et al. 2009; Spaans et al. 2023; Telzer et al. 2010; van de Groep et al. 2022), even though previous developmental studies also suggest that giving becomes more sensitive to the target (Güroğlu et al. 2014; Padilla‐Walker and Carlo 2014; van de Groep et al. 2022). Older and more pubertally matured adolescents may be more able to consider the need of the recipient, even though they do not necessarily receive something in return, which may be reflected by more advanced social‐cognitive skills such as perspective taking (Crone et al. 2022; Crone and Dahl 2012; van de Groep, Zanolie, et al. 2020).

Second, we also found that pubertal development is associated with an increase in support for friends and peers. Specifically, adolescents show more emotional support and helping behavior toward friends and peers with increasing pubertal development, independent of their age. Although we are—to our knowledge—the first to demonstrate that pubertal maturation is linked to prosocial behavior toward friends and peers, this is in line with previous studies showing age‐related increases in prosocial behaviors to friends (Güroğlu et al. 2014; Padilla‐Walker et al. 2018). However, this contrasts with the peak (i.e., increases and decreases) in prosocial behavior during mid‐to‐late adolescence found in previous cross‐sectional studies (Blankenstein et al. 2020; Sweijen et al. 2022). Although the current study did show correlations with a quadratic age pattern, indicating a peak during mid‐adolescence, differences in developmental effects may be explained by the specific parameters included. For instance, Blankenstein et al. (2020) examined the average of the different prosocial actions, and in our prior work (Sweijen et al. 2022), we focused solely on emotional support to friends, whereas in this study, we examined prosocial actions more broadly directed to both friends and peers. Building on this reasoning, the finding of a peak during mid‐adolescence when focusing solely on emotional support to friends (Sweijen et al. 2022) may suggest that friends play the most significant role in driving this peak during adolescence, especially in emotional contexts known to be more salient during adolescence as a sensitive period for affective processing (Casey et al. 2011; Crone and Dahl 2012). Notably, the age range in this study was more limited (9–22 years) compared to prior work, suggesting that possibly prosocial actions may decrease in the twenties (Blankenstein et al. 2020; Sweijen et al. 2022). Future studies should aim to replicate these patterns in support of friends and peers by differentiating between the specific type and target.

Third, for adolescents' prosocial behaviors in different situations, we showed that prosocial behavior in a public setting decreases with age, whereas dire and compliant prosocial behaviors increase across pubertal development. Regarding public prosocial behavior, the age‐related decrease may suggest that younger individuals display more rule‐based behaviors as they believe others expect it from them (Meuwese et al. 2015). Public prosocial behaviors, particularly in the presence of other people, may be driven more by social expectations, in comparison to prosocial behavior to ingroup members (e.g., friends), which may be more characterized by reciprocity (Carlo and Padilla‐Walker 2020). Furthermore, we found pubertal‐related increases in dire and compliant prosocial behavior. Same‐aged adolescents in a more developed pubertal stage may be more oriented toward the perspective and needs of the situation (Crone et al. 2022; Crone and Dahl 2012), as both dire and compliant behaviors occur in situations where action is required as a response to the needs of others. This mirrors the interpretation of increased consideration of others regarding our results on age‐ and puberty‐related increases in giving behavior. Given that we found different developmental trajectories on the basis of the situation in which the behavior may occur, this also underlines that prosocial behavior is highly dependent on the context.

Fourth, and finally, we found that some prosocial behaviors show no age‐ and pubertal‐related changes across adolescence, namely, social value orientation and altruism. This is in line with social value orientation being considered a personality‐based type of prosocial behavior, with previous studies also showing a trait‐like stability across time (Bogaert et al. 2008; Murphy et al. 2011). Although we also found stability across development for altruism, in general, and also specifically to friends and peers, we did, however, find hormone‐related decreases in altruism among females. That is, among female adolescents, altruism to friends and peers decreases as testosterone increases, suggesting that the effects of testosterone may differ for males and females. In line with our finding that testosterone potentially plays a role in weakening altruistic behaviors for females, one study also demonstrated that testosterone was negatively correlated with generosity among female adults (Novakova et al. 2024), whereas studies on male adults demonstrate that testosterone enhances altruism to ingroup members (Novakova et al. 2024; Reimers and Diekhof 2015). Our results show an interesting contribution to the existing literature on testosterone effects on prosocial behavior, as most studies suggest that testosterone is positively associated with approach‐related behaviors through elevated reward sensitivity (e.g., Peper and Dahl 2013).

Other developmental effects did not survive the correction in threshold because of multiple testing. That is, we found age and puberty effects that were only significant at an uncorrected threshold for prosocial behaviors in an anonymous (age‐related increase), emotional (age‐related peak during mid‐adolescence and puberty‐related increase), and public (puberty‐related decrease) situation, as well as for giving/sharing behavior to friends/peers (age‐related peak during mid‐ to late‐adolescence) and emotional support to friends/peers (age‐related increase). Because no conclusions on the developmental trajectories of these prosocial behaviors can be drawn on the basis of the current study, future research is needed.

### 7.2 Developmental Trajectory of Rebelliousness and Its Association With Prosocial Behavior

Regarding the development of rebelliousness, we found age‐ and puberty‐related increases in rebelliousness, but no hormone‐related changes (i.e., testosterone and estradiol). Our results are consistent with previous studies demonstrating strong age‐related increases in risk‐taking behavior (Casey 2015; Steinberg 2008), but not with other studies showing peaks in rebelliousness during mid‐adolescence, which were previously found to be driven by sex hormones (Blankenstein et al. 2020; Braams et al. 2015; Peper et al. 2018). It should be noted, however, that most studies reporting a peak in rebellious behavior included broader age ranges and report this peak around the ages of 18–20 years (Duell et al. 2018). Consistent with prior research, the developmental increase in rebelliousness was explained by both age and pubertal measures (Peper et al. 2018).

Our study provides preliminary evidence for an association between the trajectories of prosocial and rebellious behavior over time. First, for prosocial actions to friends and peers, we found positive cross‐sectional correlations with rebelliousness, similar to Blankenstein et al. (2020). Second, our longitudinal measures also showed a positive slope‐slope association, although at an uncorrected threshold; therefore, these results should be interpreted with caution. This slope‐slope association is an indication of correlated change, such that the more change in prosocial behavior, the more change in rebelliousness. Considering that the univariate models indicated a decrease in prosocial behavior and an increase in rebelliousness over time, the slope–slope association suggests that, at an individual level, when prosocial behavior decreased, rebelliousness increased over time. This association may be explained by overlapping brain regions that are implicated in both behaviors, such as reward, cognitive control, and social brain regions (Do et al. 2017). For example, increased activation in the ventral striatum was found to be associated with adolescents' prosocial risk‐taking (Telzer et al. 2013), which may be explained by increased reward sensitivity. Similarly, Blankenstein et al. (2020) demonstrated that reward drive predicted the combined profile of prosocial and risk‐taking behavior. Although the results of the current study provide evidence that rebelliousness and prosocial behavior do not develop in isolation during adolescence, more research is needed to disentangle this interaction between risk‐taking and prosociality. An interesting direction for future research is to examine distinct groups of adolescents on the basis of prosocial and risk‐taking behavior, because different behavioral typologies can be detected on the basis of the intersection of these behaviors (Do et al. 2017). Furthermore, the association with rebelliousness was only present for prosocial actions to friends and peers, but not for the other prosocial behaviors. This may be because both are concrete daily behaviors occurring in a social context with peers specifically As one can argue that risk‐taking is also a multidimensional construct and should be considered as such in analyses (e.g., Peper et al. 2018), it would be interesting if future studies also examine the interplay between different types of both risk‐taking and prosocial behavior, such as thrill seeking or positive risk‐taking.

Studies examining behaviors in a social context are also sensitive to the broader societal context in which these behaviors occur. The third wave of the current study was conducted when the COVID‐19 pandemic was adding social restrictions to youth (Orben et al. 2020). Although this is a limitation of the current study, it also provided us with the unique opportunity to disentangle effects of time and of environmental hardship caused by a global pandemic. Specifically, the LGC models on the basis of timepoints provided a suitable method for not only disentangling bivariate associations, but also general time effects, and revealed non‐linear time effects on prosocial behavior. The unexpected quadratic patterns of general time effects could therefore also reflect the impact of COVID‐19. Indeed, we included a measure of the subjective impact of COVID‐19 at the third time point, and the results revealed positive correlations between the subjective impact of COVID‐19, prosocial behavior, and rebellious behavior. In the Netherlands, in this period of data collection, adolescents partly received online education during the third lockdown, after which, when COVID‐19 infections decreased, schools were reopened, and lockdown measures were loosened and finally removed. Given that all participants experienced the restrictions of COVID‐19, we expect that age effects can be examined separately, but the results should be interpreted, as all studies, in the context of a changing world.

### 7.3 Limitations

This study has several strengths, including a longitudinal design of three waves, the preregistered analysis plan, and the consistency of measures across waves. However, several limitations should be acknowledged that future studies should address. First, larger sample sizes are needed to replicate our findings, particularly the results derived from the LGC models. Second, the female participants in our sample were mostly in the late pubertal or postpubertal category, which is, however, in line with Braams et al. (2015) demonstrating steeper increases in PDS scores for females compared to males. However, to draw more specific conclusions, future studies should aim to replicate these findings by using more balanced and larger samples of adolescents in all pubertal stages. Third, although the current study investigated the direct impact of puberty, we acknowledge that the effect of puberty mainly works through the contribution of hormones on mechanisms such as sensation seeking and reward sensitivity, which should be addressed in future research. Fourth, although the ART as a risk‐taking measure reflects contextualized behavioral expressions of rebelliousness, some age‐related differences may reflect changing opportunities rather than purely trait‐level changes. Future studies could benefit from combining behavioral indices (e.g., ART) with trait‐based measures to disentangle adolescents' propensities and opportunities.

## 8 Conclusion

Using a longitudinal design, the current study confirmed dissociable developmental trajectories of prosocial behavior during adolescence, which depend on the type, target, and situation of the behavior. That is, prosocial behaviors most likely resembling personality traits remained stable across puberty and age. Prosocial actions toward friends and peers, dire and compliant prosocial behavior, and charitable giving increased with increasing age and puberty, whereas public prosocial behavior declined with age. Our study also shows that pubertal maturation, both hormonal and physical, is an important contributor to adolescents' prosocial behavior, above and beyond age. Our study contributes to the literature by stressing the importance of taking this multidimensionality into account, as well as examining both age‐ and puberty‐related developments as both capture another developmental aspect of adolescent behavior.

## Supporting information


Table S1.

Table S2.

## Data Availability

Datasets, computer code, and materials are available at the Erasmus University Rotterdam's Data Repository upon acceptance: https://doi.org/10.25397/eur.c.7472382. The study analyses presented here were preregistered. The preregistration is available at the following URL: https://osf.io/p32jq.
