# Supplementary material for: A Longitudinal Study of Multidimensional Prosocial Behavior During Adolescence
Source: Child Dev. 2025 Jul 25;96(6):1946–67. doi: 10.1111/cdev.70009 (PMC12598452; doi:10.1111/cdev.70009)
Supplement: Supplementary file 1 — Table S1. [file CDEV-96-1946-s002.docx]

**Supplement 1**

While we followed most statistical analyses from the preregistration, we deviated from the preregistration regarding the following points. First, to describe the developmental trajectories based on age and puberty, we performed linear mixed models as preregistered alternative to LGC models. While we preregistered to use LGC models for this study aim combining TSCORES (i.e., to account for age heterogeneity at each wave) and factor loadings on the slopes (i.e., to account for the unequal intervals between waves), we discovered that fitting the slopes simultaneously on ages and timepoints is practically impossible, resulting in us not being able to follow our preregistered plan to investigate the developmental trajectories based on age. Additionally, the TSCORES models did not result in all our preregistered model fit indices (i.e., only AIC and BIC) and standardized results. We therefore decided to perform linear mixed models, with these models also being more flexible in capturing (non-)linear developmental changes using (incomplete) longitudinal data (Ghisletta et al., 2015).

To examine the association between the developments of prosocial and risk-taking behavior, we followed the preregistered LGC models using factor loadings on the slopes (see Table S3 for the models with TSCORES). That is, we examined the longitudinal trajectories of prosocial and risk-taking behaviors over time using univariate LGC models, and, subsequently, examined the bivariate associations between each prosocial and risk-taking behavior by performing bivariate LGC models. Here, we tested non-linear (i.e., freeing the slope at T2 or T3) instead of quadratic models, given that all quadratic models resulted in convergence errors. Also, we used an average score of all subscales of the OPA and PTM-R, because the preregistered second-order models could not be converged and univariate models on each subscale separately resulted in bad model fit for almost all subscales (see Table S3). Finally, for brevity and to reduce the number of analyses, we decided to not report the analyses with the interaction variable between each prosocial behavior and risk-taking behavior.

**Supplement 2**

Table S1. Linear mixed models including age and pubertal development while correcting for main and interaction effects of gender

| Measure | Model | β | SE | ANOVA | AIC | BIC |
| --- | --- | --- | --- | --- | --- | --- |
| CDG | Intercept | 4.51 | 0.83 |  | 1665.17 | 1688.93 |
|  | Linear age | 0.11 | 0.05 | *F*(1) = 8.70, *p* = .003 |  |  |
|  | Gender | -1.35 | 1.36 | *F*(1) = 9.28, *p* = .003 |  |  |
|  | Linear age * Gender | 0.03 | 0.08 | *F*(1) = 0.09, *p* = .766 |  |  |
|  | Intercept | 5.14 | 0.92 |  | 1585.91 | 1609.38 |
|  | PDS | 0.26 | 0.20 | *F*(1) = 7.43, *p* = .007 |  |  |
|  | Gender | -1.35 | 1.12 | *F*(1) = 4.01, *p* = .047 |  |  |
|  | PDS * Gender | 0.17 | 0.26 | *F*(1) = 0.39, *p* = .534 |  |  |
|  | Intercept | 4.80 | 0.94 |  | 1640.61 | 1635.96 |
|  | Linear age | 0.15 | 0.07 | *F*(1) = 6.36, *p* = .012 |  |  |
|  | PDS | -0.21 | 0.20 | *F*(1) = 0.08, *p* = .773 |  |  |
|  | Gender | -2.16 | 1.49 | *F*(1) = 8.91, *p* = .003 |  |  |
|  | Linear age * Gender | -0.03 | 0.12 | *F*(1) = 0.07, *p* = .787 |  |  |
|  | PDS * Gender | 0.43 | 0.31 | *F*(1) = 1.83, *p* = .177 |  |  |
| OPA - altruism | Intercept | 4.00 | 0.41 |  | 1068.23 | 1091.73 |
|  | Linear age | -0.04 | 0.03 | *F*(1) = 2.39, *p* = .127 |  |  |
|  | Gender | -0.97 | 0.66 | *F*(1) = 10.10, *p* = .002 |  |  |
|  | Linear age * Gender | 0.03 | 0.04 | *F*(1) = 0.64, *p* = .434 |  |  |
|  | Intercept | 2.84 | 0.39 |  | 1025.88 | 1049.15 |
|  | PDS | 0.10 | 0.09 | *F*(1) = 0.42, *p* = .518 |  |  |
|  | Gender | 0.10 | 0.50 | *F*(1) = 5.84, *p* = .017 |  |  |
|  | PDS * Gender | -0.13 | 0.12 | *F*(1) = 1.06, *p* = .304 |  |  |
|  | Intercept | 3.36 | 0.47 |  | 1035.25 | 1066.27 |
|  | Linear age | -0.06 | 0.03 | *F*(1) = 3.26, *p* = .072 |  |  |
|  | PDS | 0.22 | 0.11 | *F*(1) = 2.75, *p* = .098 |  |  |
|  | Gender | -0.24 | 0.74 | *F*(1) = 3.65, *p* = .058 |  |  |
|  | Linear age * Gender | 0.04 | 0.06 | *F*(1) = 0.55, *p* = .461 |  |  |
|  | PDS * Gender | -0.20 | 0.16 | *F*(1) = 1.52, *p* = .219 |  |  |
| OPA - emotional support | Intercept | 3.99 | 0.37 |  | 979.83 | 1003.32 |
|  | Linear age | 0.03 | 0.02 | *F*(1) = 6.29, *p* = .013 |  |  |
|  | Gender | -1.61 | 0.60 | *F*(1) = 34.36, *p* < .001 |  |  |
|  | Linear age * Gender | 0.05 | 0.04 | *F*(1) = 1.77, *p* = .184 |  |  |
|  | Intercept | 3.48 | 0.34 |  | 932.01 | 955.27 |
|  | PDS | 0.20 | 0.08 | *F*(1) = 16.91, *p* < .001 |  |  |
|  | Gender | -0.72 | 0.44 | *F*(1) = 16.41, *p* < .001 |  |  |
|  | PDS * Gender | 0.03 | 0.11 | *F*(1) = 0.08, *p* = .783 |  |  |
|  | Intercept | 3.25 | 0.42 |  | 944.07 | 975.09 |
|  | Linear age | 0.03 | 0.03 | *F*(1) = 1.62, *p* = .204 |  |  |
|  | PDS | 0.16 | 0.09 | *F*(1) = 5.18, *p* = .024 |  |  |
|  | Gender | -0.84 | 0.66 | *F*(1) = 17.76, *p* < .001 |  |  |
|  | Linear age * Gender | 0.01 | 0.05 | *F*(1) = 0.04, *p* = .851 |  |  |
|  | PDS * Gender | 0.01 | 0.14 | *F*(1) = 0.00, *p* = .953 |  |  |
| OPA - helping solve problems | Intercept | 4.71 | 0.33 |  | 936.73 | 960.23 |
|  | Linear age | 0.02 | 0.02 | *F*(1) = 0.62, *p* = .430 |  |  |
|  | Gender | -0.21 | 0.54 | *F*(1) = 10.34, *p* = .002 |  |  |
|  | Linear age * Gender | -0.01 | 0.03 | *F*(1) = 0.09, *p* = .762 |  |  |
|  | Intercept | 3.67 | 0.32 |  | 878.49 | 901.76 |
|  | PDS | 0.30 | 0.07 | *F*(1) = 15.89, *p* < .001 |  |  |
|  | Gender | 0.58 | 0.40 | *F*(1) = 1.93, *p* = .166 |  |  |
|  | PDS * Gender | -0.19 | 0.10 | *F*(1) = 3.81, *p* = .052 |  |  |
|  | Intercept | 3.88 | 0.38 |  | 889.31 | 920.33 |
|  | Linear age | -0.03 | 0.03 | *F*(1) = 2.75, *p* = .099 |  |  |
|  | PDS | 0.35 | 0.09 | *F*(1) = 19.30, *p* < .001 |  |  |
|  | Gender | 0.92 | 0.59 | *F*(1) = 0.83, *p* = .365 |  |  |
|  | Linear age * Gender | -0.03 | 0.05 | *F*(1) = 0.43, *p* = .513 |  |  |
|  | PDS * Gender | -0.13 | 0.13 | *F*(1) = 0.95, *p* = .330 |  |  |
| OPA - giving / sharing | Intercept | 3.00 | 0.36 |  | 1033.23 | 1060.64 |
|  | Linear age | 0.04 | 0.02 | *F*(1) = 3.62, *p* = .058 |  |  |
|  | Quadratic age | -0.02 | 0.01 | *F*(1) = 6.82, *p* = .010 |  |  |
|  | Gender | -0.23 | 0.21 | *F*(1) = 8.00, *p* = .005 |  |  |
|  | Quadratic age * Gender | -0.02 | 0.02 | *F*(1) = 1.19, *p* = .277 |  |  |
|  | Intercept | 2.59 | 0.37 |  | 984.04 | 1007.30 |
|  | PDS | 0.20 | 0.08 | *F*(1) = 9.00, *p* = .003 |  |  |
|  | Gender | -0.02 | 0.47 | *F*(1) = 2.13, *p* = .147 |  |  |
|  | PDS * Gender | -0.05 | 0.11 | *F*(1) = 0.20, *p* = .652 |  |  |
|  | Intercept | 2.71 | 0.48 |  | 1002.12 | 1037.02 |
|  | Linear age | 0.02 | 0.03 | *F*(1) = 0.42, *p* = .519 |  |  |
|  | Quadratic age | -0.02 | 0.01 | *F*(1) = 5.31, *p* = .023 |  |  |
|  | PDS | 0.14 | 0.10 | *F*(1) = 2.05, *p* = .153 |  |  |
|  | Gender | 0.08 | 0.55 | *F*(1) = 2.93, *p* = .089 |  |  |
|  | Quadratic age * Gender | -0.02 | 0.02 | *F*(1) = 0.78, *p* = .380 |  |  |
|  | PDS * Gender | -0.06 | 0.12 | *F*(1) = 0.21, *p* = .645 |  |  |
| PTM-R public | Intercept | 2.50 | 0.26 |  | 769.96 | 793.44 |
|  | Linear age | -0.05 | 0.02 | *F*(1) = 13.03, *p* < .001 |  |  |
|  | Gender | 0.03 | 0.43 | *F*(1) = 4.13, *p* = .044 |  |  |
|  | Linear age * Gender | 0.01 | 0.03 | *F*(1) = 0.13, *p* = .717 |  |  |
|  | Intercept | 1.99 | 0.25 |  | 736.50 | 759.77 |
|  | PDS | -0.07 | 0.06 | *F*(1) = 2.96, *p* = .086 |  |  |
|  | Gender | 0.11 | 0.32 | *F*(1) = 1.95, *p* = .165 |  |  |
|  | PDS * Gender | 0.01 | 0.08 | *F*(1) = 0.01, *p* = .941 |  |  |
|  | Intercept | 2.36 | 0.29 |  | 743.75 | 774.77 |
|  | Linear age | -0.05 | 0.02 | *F*(1) = 7.49, *p* = .007 |  |  |
|  | PDS | 0.05 | 0.07 | *F*(1) = 0.41, *p* = .523 |  |  |
|  | Gender | 0.17 | 0.46 | *F*(1) = 4.74, *p* = .031 |  |  |
|  | Linear age * Gender | 0.01 | 0.04 | *F*(1) = 0.05, *p* = .827 |  |  |
|  | PDS * Gender | -0.02 | 0.11 | *F*(1) = 0.05, *p* = .828 |  |  |
| PTM-R - anonymous | Intercept | 1.69 | 0.32 |  | 862.22 | 885.70 |
|  | Linear age | 0.02 | 0.02 | *F*(1) = 4.11, *p* = .043 |  |  |
|  | Gender | -0.20 | 0.52 | *F*(1) = 0.27, *p* = .602 |  |  |
|  | Linear age * Gender | 0.02 | 0.03 | *F*(1) = 0.27, *p* = .603 |  |  |
|  | Intercept | 2.13 | 0.29 |  | 836.92 | 860.19 |
|  | PDS | -0.01 | 0.06 | *F*(1) = 0.31, *p* = .581 |  |  |
|  | Gender | -0.20 | 0.38 | *F*(1) = 0.28, *p* = .600 |  |  |
|  | PDS * Gender | 0.07 | 0.09 | *F*(1) = 0.56, *p* = .455 |  |  |
|  | Intercept | 1.84 | 0.35 |  | 846.01 | 877.03 |
|  | Linear age | 0.04 | 0.03 | *F*(1) = 4.72, *p* = .031 |  |  |
|  | PDS | -0.08 | 0.08 | *F*(1) = 1.35, *p* = .247 |  |  |
|  | Gender | -0.55 | 0.56 | *F*(1) = 0.00, *p* = .988 |  |  |
|  | Linear age * Gender | 0.03 | 0.05 | *F*(1) = 0.40, *p* = .528 |  |  |
|  | PDS * Gender | 0.02 | 0.12 | *F*(1) = 0.03, *p* = .874 |  |  |
| PTM-R - dire | Intercept | 3.89 | 0.30 |  | 863.82 | 887.30 |
|  | Linear age | -0.01 | 0.02 | *F*(1) = 0.07, *p* = .788 |  |  |
|  | Gender | -0.66 | 0.50 | *F*(1) = 2.91, *p* = .090 |  |  |
|  | Linear age * Gender | 0.03 | 0.03 | *F*(1) = 0.94, *p* = .333 |  |  |
|  | Intercept | 3.10 | 0.28 |  | 819.76 | 843.03 |
|  | PDS | 0.15 | 0.06 | *F*(1) = 9.05, *p* = .003 |  |  |
|  | Gender | 0.10 | 0.36 | *F*(1) = 0.28, *p* = .598 |  |  |
|  | PDS * Gender | -0.04 | 0.09 | *F*(1) = 0.21, *p* = .651 |  |  |
|  | Intercept | 3.45 | 0.34 |  | 830.02 | 861.04 |
|  | Linear age | -0.05 | 0.03 | *F*(1) = 3.73, *p* = .054 |  |  |
|  | PDS | 0.25 | 0.08 | *F*(1) = 11.93, *p* = .001 |  |  |
|  | Gender | -0.04 | 0.54 | *F*(1) = 0.00, *p* = .977 |  |  |
|  | Linear age * Gender | 0.03 | 0.04 | *F*(1) = 0.32, *p* = .571 |  |  |
|  | PDS * Gender | -0.10 | 0.12 | *F*(1) = 0.60, *p* = .438 |  |  |
| PTM-R - emotional | Intercept | 3.81 | 0.30 |  | 899.25 | 926.65 |
|  | Linear age | -0.02 | 0.02 | *F*(1) = 1.45, *p* = .230 |  |  |
|  | Quadratic age | -0.02 | 0.01 | *F*(1) = 5.61, *p* = .019 |  |  |
|  | Gender | -0.18 | 0.17 | *F*(1) = 2.19, *p* = .141 |  |  |
|  | Quadratic age * Gender | 0.00 | 0.02 | *F*(1) = 0.00, *p* = .991 |  |  |
|  | Intercept | 3.20 | 0.30 |  | 860.62 | 883.89 |
|  | PDS | 0.04 | 0.07 | *F*(1) = 1.13, *p* = .288 |  |  |
|  | Gender | -0.24 | 0.39 | *F*(1) = 1.14, *p* = .288 |  |  |
|  | PDS * Gender | 0.03 | 0.09 | *F*(1) = 0.09, *p* = .769 |  |  |
|  | Intercept | 3.84 | 0.39 |  | 878.45 | 913.35 |
|  | Linear age | -0.04 | 0.02 | *F*(1) = 3.43, *p* = .065 |  |  |
|  | Quadratic age | -0.02 | 0.01 | *F*(1) = 4.69, *p* = .032 |  |  |
|  | PDS | 0.07 | 0.08 | *F*(1) = 2.52, *p* = .114 |  |  |
|  | Gender | -0.33 | 0.45 | *F*(1) = 0.47, *p* = .495 |  |  |
|  | Quadratic age * Gender | 0.01 | 0.02 | *F*(1) = 0.07, *p* = .790 |  |  |
|  | PDS * Gender | 0.05 | 0.10 | *F*(1) = 0.29, *p* = .593 |  |  |
| PTM-R - compliant | Intercept | 3.51 | 0.32 |  | 933.91 | 957.39 |
|  | Linear age | 0.02 | 0.02 | *F*(1) = 0.58, *p* = .447 |  |  |
|  | Gender | -0.01 | 0.53 | *F*(1) = 10.37, *p* = .002 |  |  |
|  | Linear age * Gender | -0.02 | 0.03 | *F*(1) = 0.42, *p* = .517 |  |  |
|  | Intercept | 3.41 | 0.31 |  | 890.65 | 913.91 |
|  | PDS | 0.10 | 0.07 | *F*(1) = 5.93, *p* = .015 |  |  |
|  | Gender | -0.42 | 0.39 | *F*(1) = 5.54, *p* = .020 |  |  |
|  | PDS * Gender | 0.04 | 0.10 | *F*(1) = 0.16, *p* = .691 |  |  |
|  | Intercept | 3.42 | 0.36 |  | 901.08 | 932.10 |
|  | Linear age | 0.00 | 0.03 | *F*(1) = 1.22, *p* = .270 |  |  |
|  | PDS | 0.10 | 0.09 | *F*(1) = 7.02, *p* = .008 |  |  |
|  | Gender | 0.27 | 0.57 | *F*(1) = 3.59, *p* = .060 |  |  |
|  | Linear age * Gender | -0.07 | 0.05 | *F*(1) = 2.30, *p* = .131 |  |  |
|  | PDS * Gender | 0.18 | 0.14 | *F*(1) = 1.75, *p* = .187 |  |  |
| PTM-R - altruism | Intercept | 4.05 | 0.24 |  | 665.81 | 689.29 |
|  | Linear age | 0.01 | 0.01 | *F*(1) = 3.11, *p* = .079 |  |  |
|  | Gender | -0.56 | 0.39 | *F*(1) = 10.64, *p* = .001 |  |  |
|  | Linear age * Gender | 0.02 | 0.02 | *F*(1) = 0.48, *p* = .490 |  |  |
|  | Intercept | 4.39 | 0.22 |  | 632.14 | 655.40 |
|  | PDS | -0.03 | 0.05 | *F*(1) = 0.07, *p* = .797 |  |  |
|  | Gender | -0.54 | 0.28 | *F*(1) = 7.75, *p* = .006 |  |  |
|  | PDS * Gender | 0.07 | 0.07 | *F*(1) = 1.09, *p* = .296 |  |  |
|  | Intercept | 4.12 | 0.26 |  | 644.09 | 675.12 |
|  | Linear age | 0.03 | 0.02 | *F*(1) = 3.00, *p* = .084 |  |  |
|  | PDS | -0.10 | 0.06 | *F*(1) = 0.97, *p* = .326 |  |  |
|  | Gender | -0.40 | 0.42 | *F*(1) = 9.75, *p* = .002 |  |  |
|  | Linear age * Gender | -0.02 | 0.03 | *F*(1) = 0.38, *p* = .536 |  |  |
|  | PDS * Gender | 0.11 | 0.09 | *F*(1) = 1.50, *p* = .221 |  |  |
| SVO | Intercept | 37.08 | 4.20 |  | 2708.60 | 2731.93 |
|  | Linear age | -0.18 | 0.26 | *F*(1) = 1.23, *p* = .269 |  |  |
|  | Gender | -2.31 | 6.80 | *F*(1) = 7.82, *p* = .006 |  |  |
|  | Linear age * Gender | -0.11 | 0.41 | *F*(1) = 0.07, *p* = .794 |  |  |
|  | Intercept | 36.21 | 4.61 |  | 2586.08 | 2609.14 |
|  | PDS | -0.54 | 1.03 | *F*(1) = 0.64, *p* = .426 |  |  |
|  | Gender | -4.07 | 5.62 | *F*(1) = 5.90, *p* = .016 |  |  |
|  | PDS * Gender | 0.02 | 1.33 | *F*(1) = 0.00, *p* = .987 |  |  |
|  | Intercept | 36.05 | 5.09 |  | 2587.97 | 2618.72 |
|  | Linear age | 0.02 | 0.38 | *F*(1) = 0.51, *p* = .474 |  |  |
|  | PDS | -0.59 | 1.37 | *F*(1) = 0.02, *p* = .901 |  |  |
|  | Gender | 2.58 | 7.78 | *F*(1) = 4.44, *p* = .036 |  |  |
|  | Linear age * Gender | -0.70 | 0.64 | *F*(1) = 1.19, *p* = .275 |  |  |
|  | PDS * Gender | 1.34 | 1.87 | *F*(1) = 0.50, *p* = .479 |  |  |
| ART | Intercept | -3.36 | 0.38 |  | 832.39 | 855.40 |
|  | Linear age | 0.27 | 0.02 | *F*(1) = 220.69, *p* < .001 |  |  |
|  | Gender | 0.40 | 0.61 | *F*(1) = 0.44, *p* = .510 |  |  |
|  | Linear age * Gender | -0.02 | 0.04 | *F*(1) = 0.26, *p* = .609 |  |  |
|  | Intercept | -1.38 | 0.44 |  | 937.63 | 960.58 |
|  | PDS | 0.57 | 0.10 | *F*(1) = 62.27, *p* < .001 |  |  |
|  | Gender | 0.96 | 0.54 | *F*(1) = 8.63, *p* = .004 |  |  |
|  | PDS * Gender | -0.11 | 0.13 | *F*(1) = 0.78, *p* = .377 |  |  |
|  | Intercept | 36.05 | 5.09 |  | 2587.97 | 2618.72 |
|  | Linear age | 0.03 | 0.38 | *F*(1) = 0.51, *p* = .474 |  |  |
|  | PDS | -0.59 | 1.37 | *F*(1) = 0.02, *p* = .901 |  |  |
|  | Gender | 2.58 | 7.78 | *F*(1) = 4.44, *p* = .037 |  |  |
|  | Linear age * Gender | -0.70 | 0.64 | *F*(1) = 1.19, *p* = .275 |  |  |
|  | PDS * Gender | 1.34 | 1.87 | *F*(1) = 0.50, *p* = .478 |  |  |

*Note*. AIC = Akaike Information Criteria; BIC = Bayesian Information Criterion; PDS = Pubertal Development Scale; CDG = Charity Dictator Game; OPA = Opportunities for Prosocial Actions; PTM-R = Prosocial Tendencies Measure – Revised; SVO = Social Value Orientation; ART = Adolescent Risk-Taking Questionnaire.

Table S3. Model fit indices for models using TSCORES

|  | Prosocial | | | | | | | | | | | | Risk-taking | | |  |
| --- | --- | --- | --- | --- | --- | --- | --- | --- | --- | --- | --- | --- | --- | --- | --- | --- |
|  | CDG | | OPA | | | PTM-R | | | SVO | | | ART | | | |  |
|  | AIC | BIC | | AIC | BIC | | AIC | BIC | | AIC | BIC | | | AIC | BIC | |
| Intercept only | 1665.08 | 1679.86 | | 871.21 | 885.99 | | 394.44 | 409.15 | | 2717.32 | 2731.99 | | | 963.51 | 978.21 | |
| Linear with fixed slopes | 1653.77 | 1674.46 | | 879.12 | 899.81 | | 426.04 | 446.63 | | 2720.59 | 2741.13 | | | 770.84 | 791.43 | |
| Linear with random slopes | 1652.88 | 1676.53 | | 875.88 | 899.52 | | 400.29 | 423.83 | | 2720.58 | 2744.05 | | | 771.07 | 794.60 | |
| Quadratic with fixed slopes | 1657.83 | 1690.34 | | 880.75 | 913.26 | | 403.65 | 436.01 | | 2739.04 | 2771.32 | | | ^a^ | ^a^ | |
| Quadratic with random slopes | 1646.77 | 1682.24 | | 882.48 | 917.95 | | 404.38 | 439.68 | | 2721.72 | 2756.93 | | | ^a^ | ^a^ | |

*Note.* AIC = Akaike Information Criteria; BIC = Bayesian Information Criterion; CDG = Charity Dictator Game; OPA = Opportunities for Prosocial Actions; PTM-R = Prosocial Tendencies Measure – Revised; SVO = Social Value Orientation; ART = Adolescent Risk-Taking Questionnaire

^a^ Model fit indices could not be computed due to convergence problems

Table S4. Model fit indices for models on each subscale of OPA and PTM-R.

|  | OPA - altruism | | | | | OPA – emotional support | | | | |
| --- | --- | --- | --- | --- | --- | --- | --- | --- | --- | --- |
|  | χ^2^ | RMSEA [95% CI] | CFI | AIC | BIC | χ^2^ | RMSEA [95% CI] | CFI | AIC | BIC |
| Intercept only | *p* < .001 | 0.28 [0.21, 0.35] | 0.420 | 1062.34 | 1077.12 | *p* < .001 | 0.20 [0.13, 0.27] | 0.776 | 998.76 | 1013.54 |
| Linear with fixed slopes | *p* < .001 | 0.45 [0.38, 0.52] | 0.000 | 1145.96 | 1160.74 | *p* < .001 | 0.45 [0.38, 0.52] | 0.000 | 1111.19 | 1125.97 |
| Linear with random slopes | *p* < .001 | 0.49 [0.36, 0.64] | 0.552 | 1058.65 | 1082.30 | *p* < .001 | 0.39 [0.26, 0.53] | 0.788 | 995.92 | 1019.57 |
| Non-linear with fixed slopes; freeing slope loading of T2 | *p* < .001 | 0.50 [0.42, 0.58] | 0.000 | 1147.95 | 1165.69 | *p* < .001 | 0.52 [0.44, 0.60] | 0.000 | 1113.08 | 1130.81 |
| Non-linear with fixed slopes; freeing slope loading of T3 | *p* < .001 | 0.31 [0.23, 0.39] | 0.473 | 1064.49 | 1082.23 | *p* < .001 | 0.33 [0.26, 0.42] | 0.529 | 1033.82 | 1051.55 |
| Non-linear with random slopes; freeing slope loading of T2 | ^a^ | ^a^ | ^a^ | ^a^ | ^a^ | ^a^ | ^a^ | ^a^ | ^a^ | ^a^ |
| Non-linear with random slopes; freeing slope loading of T3 | ^a^ | ^a^ | ^a^ | ^a^ | ^a^ | ^a^ | ^a^ | ^a^ | ^a^ | ^a^ |
|  | OPA – helping | | | | | OPA – giving/sharing | | | | |
| Intercept only | *p* = .001 | 0.16 [0.09, 0.23] | 0.596 | 920.58 | 935.36 | *p* < .001 | 0.22 [0.15, 0.29] | 0.619 | 1024.73 | 1039.51 |
| Linear with fixed slopes | *p* < .001 | 0.46 [0.39, 0.53] | 0.000 | 1064.52 | 1079.30 | *p* < .001 | 0.48 [0.40, 0.56] | 0.000 | 1130.08 | 1147.82 |
| Linear with random slopes | *p* < .001 | 0.33 [0.20, 0.48] | 0.551 | 921.78 | 945.42 | *p* < .001 | 0.45 [0.32, 0.60] | 0.580 | 1028.92 | 1052.57 |
| Non-linear with fixed slopes; freeing slope loading of T2 | *p* < .001 | 0.57 [0.49, 0.65] | 0.000 | 1066.36 | 1084.09 | *p* < .001 | 0.58 [0.49, 0.68] | 0.000 | 1126.53 | 1147.22 |
| Non-linear with fixed slopes; freeing slope loading of T3 | *p* < .001 | 0.30 [0.21, 0.41] | 0.233 | 954.61 | 975.30 | *p* < .001 | 0.35 [0.28, 0.44] | 0.235 | 1061.27 | 1079.00 |
| Non-linear with random slopes; freeing slope loading of T2 | ^a^ | ^a^ | ^a^ | ^a^ | ^a^ | ^a^ | ^a^ | ^a^ | ^a^ | ^a^ |
| Non-linear with random slopes; freeing slope loading of T3 | ^a^ | ^a^ | ^a^ | ^a^ | ^a^ | ^a^ | ^a^ | ^a^ | ^a^ | ^a^ |
|  | PTM-R public | | | | | PTM-R anonymous | | | | |
| Intercept only | *p* = .009 | 0.13 [0.06, 0.21] | 0.531 | 760.02 | 774.73 | *p* < .001 | 0.17 [0.10, 0.24] | 0.809 | 844.65 | 859.36 |
| Linear with fixed slopes | *p* < .001 | 0.52 [0.44, 0.60] | 0.000 | 951.40 | 969.05 | *p* < .001 | 0.55 [0.47, 0.63] | 0.000 | 1001.02 | 1018.67 |
| Linear with random slopes | ^a^ | ^a^ | ^a^ | ^a^ | ^a^ | *p* = .927 | 0.00 [0.00, 0.06] | 1.000 | 830.26 | 850.85 |
| Non-linear with fixed slopes; freeing slope loading of T2 | *p* < .001 | 0.64 [0.55, 0.74] | 0.000 | 956.09 | 976.68 | *p* < .001 | 0.68 [0.58, 0.78] | 0.000 | 994.95 | 1015.54 |
| Non-linear with fixed slopes; freeing slope loading of T3 | *p* < .001 | 0.41 [0.32, 0.51] | 0.000 | 831.68 | 852.27 | *p* < .001 | 0.60 [0.51, 0.71] | 0.000 | 928.22 | 948.81 |
| Non-linear with random slopes; freeing slope loading of T2 | ^a^ | ^a^ | ^a^ | ^a^ | ^a^ | *p* = .672 | 0.00 [0.00, 0.17] | 1.000 | 832.26 | 855.79 |
| Non-linear with random slopes; freeing slope loading of T3 | ^a^ | ^a^ | ^a^ | ^a^ | ^a^ | ^a^ | ^a^ | ^a^ | ^a^ | ^a^ |
|  | PTM-R dire | | | | | PTM-R emotional | | | | |
| Intercept only | *p* = .035 | 0.11 [0.03, 0.19] | 0.843 | 847.34 | 862.05 | *p* = .382 | 0.02 [0.00, 0.13] | 0.995 | 869.14 | 883.84 |
| Linear with fixed slopes | *p* < .001 | 0.52 [0.44, 0.60] | 0.000 | 993.24 | 1010.89 | *p* < .001 | 0.58 [0.50, 0.66] | 0.000 | 1055.38 | 1073.03 |
| Linear with random slopes | *p* = .822 | 0.00 [0.00, 0.14] | 1.000 | 843.62 | 867.15 | *p* = .192 | 0.07 [0.00, 0.25] | 0.982 | 872.37 | 895.91 |
| Non-linear with fixed slopes; freeing slope loading of T2 | *p* < .001 | 0.64 [0.54, 0.74] | 0.000 | 995.10 | 1015.69 | *p* < .001 | 0.70 [0.60, 0.80] | 0.000 | 1044.90 | 1065.50 |
| Non-linear with fixed slopes; freeing slope loading of T3 | *p* < .001 | 0.38 [0.29, 0.49] | 0.000 | 896.04 | 916.64 | *p* < .001 | 0.35 [0.26, 0.46] | 0.110 | 935.66 | 956.25 |
| Non-linear with random slopes; freeing slope loading of T2 | ^a^ | ^a^ | ^a^ | ^a^ | ^a^ | ^a^ | ^a^ | ^a^ | ^a^ | ^a^ |
| Non-linear with random slopes; freeing slope loading of T3 | ^a^ | ^a^ | ^a^ | ^a^ | ^a^ | ^a^ | ^a^ | ^a^ | ^a^ | ^a^ |
|  | PTM-R compliant | | | | | PTM-R altruism | | | | |
| Intercept only | *p* = .575 | 0.00 [0.00, 0.11] | 1.000 | 924.67 | 939.38 | *p* = .605 | 0.00 [0.00, 0.11] | 1.000 | 649.69 | 664.40 |
| Linear with fixed slopes | *p* < .001 | 0.48 [0.40, 0.56] | 0.000 | 1051.25 | 1068.90 | *p* < .001 | 0.51 [0.44, 0.60] | 0.000 | 877.54 | 895.19 |
| Linear with random slopes | *p* = .470 | 0.00 [0.00, 0.20] | 1.000 | 927.94 | 951.47 | *p* = .862 | 0.00 [0.00, 0.12] | 1.000 | 652.97 | 676.50 |
| Non-linear with fixed slopes; freeing slope loading of T2 | *p* < .001 | 0.59 [0.50, 0.69] | 0.000 | 1050.31 | 1070.90 | *p* < .001 | 0.67 [0.58, 0.77] | 0.000 | 879.12 | 899.71 |
| Non-linear with fixed slopes; freeing slope loading of T3 | *p* < .001 | 0.29 [0.20, 0.39] | 0.000 | 968.93 | 989.53 | *p* < .001 | 3.04 [2.94, 3.14] | 0.000 | 783.60 | 804.19 |
| Non-linear with random slopes; freeing slope loading of T2 | ^a^ | ^a^ | ^a^ | ^a^ | ^a^ | *p* < .001 | 0.00 [0.00, 0.00] | 1.000 | 654.94 | 681.41 |
| Non-linear with random slopes; freeing slope loading of T3 | ^a^ | ^a^ | ^a^ | ^a^ | ^a^ | ^a^ | ^a^ | ^a^ | ^a^ | ^a^ |

*Note.* AIC = Akaike Information Criteria; BIC = Bayesian Information Criterion; OPA = Opportunities for Prosocial Actions; PTM-R = Prosocial Tendencies Measure – Revised

^a^ Model fit indices could not be computed due to convergence problems

Table S5. Model fit indices of LGC models with prosocial behavior

|  | CDG | | | | | OPA | | | | |
| --- | --- | --- | --- | --- | --- | --- | --- | --- | --- | --- |
|  | χ^2^ | RMSEA [95% CI] | CFI | AIC | BIC | χ^2^ | RMSEA [95% CI] | CFI | AIC | BIC |
| Intercept only | *p* < .001 | 0.25 [0.19, 0.33] | 0.468 | 1665.08 | 1679.86 | *p* < .001 | 0.26 [0.20, 0.34] | 0.571 | 871.21 | 885.99 |
| Linear with fixed slopes | *p* = .109 | 0.09 [0.00, 0.21] | 0.965 | 1629.36 | 1650.05 | *p* < .001 | 0.52 [0.45, 0.59] | 0.000 | 1016.60 | 1031.38 |
| Linear with random slopes | *p* = .054 | 0.14 [0.00, 0.30] | 0.961 | 1629.88 | 1653.53 | *p* < .001 | 0.55 [0.42, 0.69] | 0.533 | 870.99 | 894.64 |
| Non-linear with fixed slopes; freeing slope loading of T2 | *p* = .204 | 0.06 [0.00, 0.19] | 0.983 | 1628.02 | 1648.71 | *p* < .001 | 0.58 [0.50, 0.66] | 0.000 | 1018.26 | 1036.00 |
| Non-linear with fixed slopes; freeing slope loading of T3 | *p* = .088 | 0.12 [0.00, 0.28] | 0.972 | 1628.01 | 1651.66 | *p* < .001 | 0.41 [0.33, 0.49] | 0.231 | 923.02 | 940.76 |
| Non-linear with random slopes; freeing slope loading of T2 | *p* = .461 | 0.00 [0.00, 0.20] | 1.000 | 1627.20 | 1650.85 | ^a^ | ^a^ | ^a^ | ^a^ | ^a^ |
| Non-linear with random slopes; freeing slope loading of T3 | ***p* = .461** | **0.00 [0.00, 0.20]** | **1.000** | **1627.2** | **1650.85** | ***p* = .207** | **0.07 [0.00, 0.24]** | **0.993** | **835.48** | **859.12** |
|  | PTM-R | | | | | SVO | | | | |
| Intercept only | ***p* = .691** | **0.00 [0.00, 0.10]** | **1.000** | **394.44** | **409.15** | p = .029 | 0.11 [0.03, 0.19] | 0.740 | 2717.32 | 2731.99 |
| Linear with fixed slopes | ^a^ | ^a^ | ^a^ | ^a^ | ^a^ | p = .011 | 0.16 [0.07, 0.27] | 0.732 | 2719.83 | 2740.37 |
| Linear with random slopes | *p* = .776 | 0.00 [0.00, 0.15] | 1.000 | 398.27 | 421.80 | **p = .315** | **0.01 [0.00, 0.23]** | **1.000** | **2713.76** | **2737.24** |
| Non-linear with fixed slopes; freeing slope loading of T2 | *p* < .001 | 1.03 [0.93, 1.13] | 0.000 | 712.44 | 733.03 | ^a^ | ^a^ | ^a^ | ^a^ | ^a^ |
| Non-linear with fixed slopes; freeing slope loading of T3 | *p* < .001 | 0.57 [0.47, 0.67] | 0.000 | 579.35 | 599.95 | ^a^ | ^a^ | ^a^ | ^a^ | ^a^ |
| Non-linear with random slopes; freeing slope loading of T2 | ^a^ | ^a^ | ^a^ | ^a^ | ^a^ | ^a^ | ^a^ | ^a^ | ^a^ | ^a^ |
| Non-linear with random slopes; freeing slope loading of T3 | ^a^ | ^a^ | ^a^ | ^a^ | ^a^ | ^a^ | ^a^ | ^a^ | ^a^ | ^a^ |

*Note.* AIC = Akaike Information Criteria; BIC = Bayesian Information Criterion; CDG = Charity Dictator Game; OPA = Opportunities for Prosocial Actions; PTM-R = Prosocial Tendencies Measure – Revised; SVO = Social Value Orientation; ART = Adolescent Risk-Taking Questionnaire. Bold values indicate best fitting model.

^a^ Model fit indices could not be computed due to convergence problems

Table S6. Model fit indices of LGC model with risk-taking behavior (ART)

|  | χ^2^ | RMSEA [95% CI] | CFI | AIC | BIC |
| --- | --- | --- | --- | --- | --- |
| Intercept only | *p* < .001 | 0.36 [0.29, 0.43] | 0.300 | 963.51 | 978.21 |
| Linear with fixed slopes | *p* < .001 | 0.33 [0.26, 0.40] | 0.411 | 982.42 | 997.13 |
| Linear with random slopes | ***p* = .182** | **0.07 [0.00, 0.17]** | **0.982** | **873.68** | **891.33** |
| Non-linear with fixed slopes by freeing slope loading of T2 | *p* < .001 | 0.39 [0.31, 0.47] | 0.390 | 977.67 | 995.32 |
| Non-linear with fixed slopes by freeing slope loading of T3 | *p* < .001 | 0.33 [0.25, 0.41] | 0.554 | 971.48 | 989.13 |
| Non-linear with random slopes by freeing slope loading of T2 | *p* = .009 | 0.20 [0.08, 0.36] | 0.944 | 874.99 | 898.52 |
| Non-linear with random slopes by freeing slope loading of T3 | *p* = .009 | 0.20 [0.08, 0.36] | 0.944 | 874.99 | 898.52 |

*Note.* AIC = Akaike Information Criteria; BIC = Bayesian Information Criterion. Bold values indicate best fitting model.

Table S7. Model fit indices of the bivariate LGC models between risk-taking and prosocial behavior

|  | χ^2^ | RMSEA [95% CI] | CFI | AIC | BIC |
| --- | --- | --- | --- | --- | --- |
| ART with CDG | χ^2^(9) = 9.84, *p* = .364 | 0.03 [0.00, 0.10] | 0.997 | 2507.72 | 2560.93 |
| ART with OPA | χ^2^(9) = 13.49, *p* = .142 | 0.06 [0.00, 0.12] | 0.984 | 1702.18 | 1775.38 |
| ART with PTM-R | χ^2^(14) = 19.48, *p* = .147 | 0.05 [0.00, 0.10] | 0.975 | 1271.84 | 1310.18 |
| ART with SVO | χ^2^(9) = 17.36, *p* = .043 | 0.08 [0.01, 0.14] | 0.958 | 3592.74 | 3645.81 |

*Note.* AIC = Akaike Information Criteria; BIC = Bayesian Information Criterion; CDG = Charity Dictator Game; OPA = Opportunities for Prosocial Actions; PTM-R = Prosocial Tendencies Measure – Revised; SVO = Social Value Orientation; ART = Adolescent Risk-Taking Questionnaire. Best-fitting models for each measure were used in the bivariate LGC models. ART: linear model with random slope with the variances of T1 and T3 fixed to 0. CDG: non-linear model with random slope with variance of T1 fixed to 0 and the slope loading of T3 freely estimated. OPA: non-linear model with random slopes with the variance of T2 fixed to 0 and the slope loading of T3 freely estimated. PTM-R: intercept only model. SVO: linear model with random slope.
